# BAK1 is involved in AtRALF1-induced inhibition of root cell expansion

**DOI:** 10.1371/journal.pgen.1007053

**Published:** 2017-10-13

**Authors:** Keini Dressano, Paulo H. O. Ceciliato, Aparecida L. Silva, Juan Carlos Guerrero-Abad, Tábata Bergonci, Fausto Andrés Ortiz-Morea, Marco Bürger, Marcio C. Silva-Filho, Daniel S. Moura

**Affiliations:** 1 Laboratório de Bioquímica de Proteínas, Departamento de Ciências Biológicas, Escola Superior de Agricultura Luiz de Queiroz, Universidade de São Paulo (ESALQ/USP), Piracicaba, SP, Brazil; 2 Plant Biology Laboratory, The Salk Institute for Biological Studies, La Jolla, California, United States of America; 3 Laboratório de Biologia Molecular de Plantas, Departamento de Genética, Escola Superior de Agricultura Luiz de Queiroz, Universidade de São Paulo (ESALQ/USP), Piracicaba, SP, Brazil; National University of Singapore and Temasek Life Sciences Laboratory, SINGAPORE

## Abstract

The rapid alkalinization factor (RALF) peptide negatively regulates cell expansion, and an antagonistic relationship has been demonstrated between AtRALF1, a root-specific RALF isoform in *Arabidopsis*, and brassinosteroids (BRs). An evaluation of the response of BR signaling mutants to AtRALF1 revealed that BRI1-associated receptor kinase1 (*bak1*) mutants are insensitive to AtRALF1 root growth inhibition activity. BAK1 was essential for the induction of AtRALF1-responsive genes but showed no effect on the mobilization of Ca^2+^ and alkalinization responses. Homozygous plants accumulating AtRALF1 and lacking the *BAK1* gene did not exhibit the characteristic semi-dwarf phenotype of AtRALF1-overexpressors. Biochemical evidence indicates that AtRALF1 and BAK1 physically interact with a K_d_ of 4.6 μM and acridinium-labeled AtRALF1 was used to demonstrate that part of the specific binding of AtRALF1 to intact seedlings and to a microsomal fraction derived from the roots of *Arabidopsis* plants is BAK1-dependent. Moreover, AtRALF1 induces an increase in BAK1 phosphorylation, suggesting that the binding of AtRALF1 to BAK1 is functional.

These findings show that BAK1 contains an additional AtRALF1 binding site, indicating that this protein may be part of a AtRALF1-containing complex as a co-receptor, and it is required for the negative regulation of cell expansion.

## Author summary

The rapid alkalinization factor (RALF) is a small peptide hormone that binds to FERONIA receptor and inhibits cell expansion. The AtRALF1, a root-specific RALF isoform in *Arabidopsis*, opposes brassinosteroid (BR) effects on root cell elongation. To address the role of AtRALF1 and BRs in regulating cell expansion, we evaluated the response of BR signaling mutants to AtRALF1. We found that the BRI1-associated receptor kinase1 (*bak1*) mutants are insensitive to AtRALF1 root growth inhibition activity. Although BAK1 showed no effect on the mobilization of Ca^2+^ and alkalinization responses, it was required for the induction of AtRALF1-responsive genes and for the semi-dwarf phenotype of AtRALF1-overexpressing plants. Different experiments demonstrated that AtRALF1 and BAK1 interact physically in a specific manner. We also suggested that the binding of AtRALF1 to BAK1 is functional because AtRALF1 induces an increase in BAK1 phosphorylation. Our findings indicate that BAK1 plays an important role in AtRALF1 signaling.

## Introduction

Rapid alkalinization factor (RALF) is a small peptide hormone first isolated from the leaves of tobacco plants that rapidly increases the pH of the extracellular media [1]. The *RALF* gene encodes a preproprotein with an N-terminal secretion signal and a conserved C-terminal sequence that harbors the active peptide [1]. When the tobacco RALF precursor was fused to green fluorescent protein, it could be detected first in the endoplasmic reticulum and then in the apoplast [2]. The RALF precursor is processed by convertases at a conserved dibasic site located upstream of the active peptide [3,4].

RALF is ubiquitous in the plant kingdom, and the model plant *Arabidopsis* contains a family of 37 isoforms of RALF peptides (AtRALFs) [5]. The peptide hormone RALF inhibits root growth by negatively regulating cell expansion [6,7]. Plants overexpressing the *AtRALF1*, *AtRALF8* and *AtRALF23* genes are semi-dwarf and have shorter roots [3,4,8]. Plants with partial silencing or knockout of the *AtRALF1* isoform have longer roots [9, 10]. RALF peptides have also been associated with the activation of a MAP kinase and the mobilization of calcium [1,5,11]. RALF peptides have also been found in *Fusarium oxysporum*, a fungal pathogen, that uses the peptide to induce alkalinization and increase infection [12].

FERONIA (FER), a receptor-like kinase with an extracellular malectin-like domain, has been identified as the receptor of the root-specific isoform AtRALF1 [10]. LORELEI (LRE) and LRE-like GPI-AP1 (LLG1) are two glycosylphosphatidylinositol-anchored proteins found in ovules and vegetative tissues, respectively, that physically interact with FER and are required for FER delivery to the cell membrane and FER signaling [13]. Recently, a downstream receptor-like cytoplasmic kinase, RPM1-induced protein kinase (RIPK) was identified as a FER interacting protein that is recruited upon AtRALF1 binding to FER [14]. The FERONIA mutant *fer4* is insensitive to low concentrations of the AtRALF1 peptide, and in this mutant, the binding of ^125^I-labeled AtRALF1 is reduced by approximately 40%. These results indicate the existence of additional AtRALF1 membrane-binding proteins. In tomato, tobacco and alfalfa cell suspension cultures, two plasma membrane proteins of 25 kDa and 120 kDa have been shown to bind to the ^125^I-azido-RALF peptide [15], and in *Arabidopsis* cell suspension cultures, the alkalinization response after sequential additions of saturating doses of RALF peptides suggests the existence of multiple RALF receptors [5].

AtRALF1-overexpressing plants are less sensitive to exogenous brassinosteroids (BRs) [9]. When treated with brassinolide (BL), plants overexpressing the AtRALF1 gene show no increase in root length, a minor increase in hypocotyl elongation and no change in the number of lateral roots [9]. AtRALF1 also induces the cytochrome P450 monooxygenase genes *Constitutive Photomorphism and Dwarfism* (*CPD*) and *DWARF4* (*DWF4*), two genes that are involved in the BR biosynthetic pathway and are down-regulated by BL treatment [9]. The *expansin* A5 gene (*AtEXPA5*), which is involved in the regulation of cell expansion in roots and is induced by BL, is repressed in the roots of AtRALF1-treated seedlings [16–18]. The *AtRALF23* gene has also been shown to be down-regulated by treating *Arabidopsis* seedlings with BL, and overexpression of the *AtRALF23* gene impairs BL-induced hypocotyl elongation [4,19].

BRs are important regulators of growth and differentiation in plants [20]. BRs bind to the extracellular domain of BR-insensitive 1 (BRI1), a leucine-rich repeat (LRR) receptor-like kinase (RLK), and induce a rapid association between BRI1 and its co-receptor BRI1-associated receptor kinase 1 (BAK1) [21,22]. BAK1 is shared by other signaling pathways and integrates several cell responses related to growth and development, as well as the innate immune system. BAK1 associates with LRR-RLK FLS2, EFR and AtPEPR1/2 [23–25]. Recently, two new roles for BAK1 have been proposed. BAK1 interacts with the tyrosine-sulfated peptide receptor PSKR1 establishing a functional complex [26] and BAK1 participates in the stomatal development through the formation of complexes with ERECTA induced by EPIDERMAL PATTERNING FACTORS (EPFs) [27].

To gather information regarding the interplay between AtRALF1 and BRs, we evaluated the responses of different mutants and transgenic *Arabidopsis* plants related to the BR pathway to the exogenous AtRALF1 treatment. We found that the primary roots of *bak1* mutants were insensitive to AtRALF1 treatment. BAK1 is required for the semi-dwarf phenotype of AtRALF1-overexpressing plants and for the induction of AtRALF1-responsive genes. However, BAK1 is not required for the AtRALF1-induced mobilization of Ca^2+^ and alkalinization. We further demonstrated that AtRALF1 physically interacts with BAK1 and that the interaction has a dissociation constant of 4.6 μM. Finally, an acridinium-labeled AtRALF1 peptide was used to show that the binding of AtRALF1 to BAK1 is specific and saturable. Our results indicate that BAK1 is an additional AtRALF1 binding site that may be part of a RALF1-containing complex. Together, these findings provide evidence that BAK1 is essential for the AtRALF1-induced inhibition of cell expansion.

## Results

### Roots of BAK1 T-DNA insertion mutants are insensitive to AtRALF1

**Fig 1:**
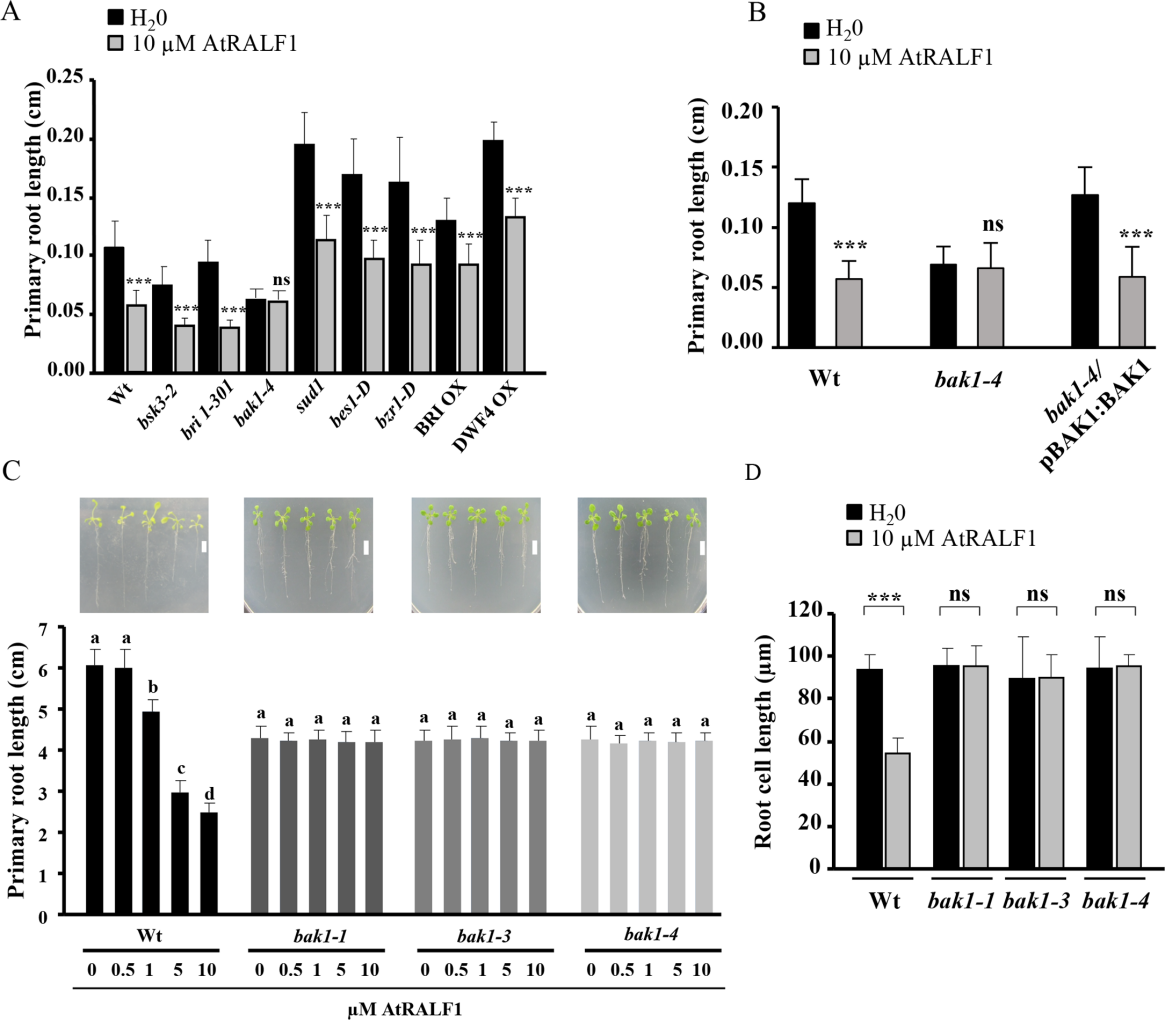
Loss-of-function *bak1* mutants are insensitive to the inhibitory activity of AtRALF1 on root growth. (A) and (B) *Arabidopsis* seedlings (2 days old) were treated with 10 μM AtRALF1 or water (H_2_0) for 2 days. The values are the mean ± SD of at least 25 seedlings. Triple asterisks indicate P < 0.01 (Student’s t test); ns, not significant. (C) Four-day-old seedlings were transferred to media containing different concentrations of AtRALF1, and the primary root length was measured after 8 days of treatment. The data represent the mean value ± SD of 30 seedlings. The means with the same letter are not significantly different from each other (Tukey’s test, P ≤ 0.01). Representative *Arabidopsis* seedlings after 8 days of treatment with AtRALF1 are shown below. Seedlings were arranged on plates after the treatment for imaging. Scale bars, 1 cm. (D) Two-day-old seedlings were treated with 10 μM AtRALF1 or water for 2 days, and root cell length was measured after 2 days of treatment. Cells from the root endodermis in the differentiation zone were measured. The values are the mean ± SD of approximately 150 cells. Triple asterisks indicate P < 0.01 (Student’s t test); ns, not significant.

To better understand the role of AtRALF1 and BRs in regulating cell expansion, we exposed different mutants and transgenic *Arabidopsis* plants with altered BR signaling pathways to the peptide hormone AtRALF1 at concentrations that inhibit root growth. Loss-of-function mutants (*bri1-301*, *bsk3-2* and *bak1-4*), gain-of-function mutants (*sud1*, *bes1-D* and *bzr1-D*) and two overexpressor lines (BRI OX and DWF4 OX) were used in the root growth assays in the presence or absence of 10 μM AtRALF1 (Fig 1A). Except for *bak1-4*, all other mutants and transgenic lines tested exhibited root growth inhibition by AtRALF1 (Fig 1A). Complementation of the *bak1-4* mutant through the genetic transformation of *bak1-4*-mutant plants with a construct harboring the BAK1 promoter and coding region (pBAK1:BAK1) recovered the sensitivity to the inhibitory effect of AtRALF1 treatment (Fig 1B). BAK1, also known as SERK3, is a member of a five-gene family in *Arabidopsis* called SOMATIC EMBRYOGENESIS RECEPTOR KINASE (SERK) [28]. To investigate whether other SERK members would also be insensitive to AtRALF1, we exposed seedlings of the mutant lines of the other four members of the SERK family and two other mutant lines of BAK1 to the peptide. AtRALF1 inhibited the root growth of *serk1-*, *serk2-*, *serk4* (*bak7*)- and *serk5* (*bak8*)-mutant seedlings but exhibited no effect on *bak1-1-* and *bak1-3-*mutant seedlings (S1 Fig). We also tested whether the insensitivity of *bak1* mutants was specific to AtRALF1. For that purpose, we used AtRALF34, a homolog of AtRALF1 with ubiquitous expression in *Arabidopsis*. AtRALF23 and AtRALF34 inhibits the primary root growth of wild-type *Arabidopsis* plants and alkalinizes the extracellular medium of cell suspensions like AtRALF1 [5]; however, unlike AtRALF1, AtRALF23 and AtRALF34 inhibited the primary root growth of the *bak1-1*, *bak1-3* and *bak1-4* mutants (S2 Fig). Two inactive AtRALF1 peptides, AtRALF1(9–49), which has a deletion of the first 8 amino acids, and AtRALF1(I6A), which has an Ile replaced by an Ala in the conserved YISY domain were used as negative controls (S2 Fig). BAK1-mutant roots normally grow up to 86% of the length of wild-type roots [21]. To maximize *bak1* root growth and to reliably evaluate the lack of inhibition of root growth, mutant seedlings were grown in vertical plates in the presence or absence of AtRALF1, and the root growth was evaluated at a later time point (after 8 days of treatment). In these experimental conditions, the results also showed that the *bak1-1*, *bak1-3 and bak1-4* mutants were insensitive to the AtRALF1 peptide, in contrast to wild-type plants (Fig 1C). Representative images of seedlings of each genotype were captured at the end of the experiment and are shown at the top of the graph. All *bak1* mutants were re-evaluated for the insertion and absence of *BAK1* gene expression (S3 Fig). Daily monitoring of the root growth during the 8-day treatment showed that by the third day of treatment, significant differences in the root growth of wild-type seedlings were already visible at doses of 1 μM and above (S4 Fig). No differences were observed in the daily root growth of the *bak1*-mutant lines. The closest homolog of BAK1 is BKK1/AtSERK4/BAK7 [29]. When the same experiment was performed using a *bak7* mutant, AtRALF1 inhibited primary root growth, demonstrating that the insensitivity is specifically associated with the absence of BAK1 (S5 Fig). To demonstrate that other root growth inhibitors would inhibit the growth of the primary root of *bak1* mutants and that our growth conditions would allow us to detect the inhibitory effect, we used the cytokinin 6-benzylaminopurine (BAP) and the auxin 1-naphthaleneacetic acid (NAA), two hormones known to inhibit primary root growth. Both the auxin and cytokinin were capable of inhibiting the root growth of *bak1* mutants (S6 and S7 Figs). Reduced sensitivity to BL was also demonstrated in our *bak1* mutants (S8 Fig). In addition, we performed a side-by-side experiment with *fer4* and the *bak1* mutants to demonstrate that, in our conditions, the *fer4* mutant reproduces the conditional insensitivity to AtRALF1 treatment, whereas *bak1* mutants are insensitive to any tested concentrations of the peptide (S9 Fig). AtRALF1 inhibits root growth by negatively regulating cell expansion [3,6,9,10]. We next investigated whether the length of *bak1* root cells was affected by AtRALF1 treatment. Endodermic root cells from the differentiation zone were evaluated because of the high level of endogenous *AtRALF1* gene expression in this layer [30,31]. Wild-type root cells from plants treated with 10 μM AtRALF1 were 58% smaller than cells from water-treated control plants, whereas the length of root cells from *bak1-1*, *bak1-3* and *bak1-4* seedlings showed no difference between AtRALF1-treated plants and the water-treated control (Fig 1D). Altogether, our findings demonstrate that plants lacking the *BAK1* gene are insensitive to the inhibition of cell expansion induced by AtRALF1 and that the insensitivity is specific to the AtRALF1 peptide.

### *bak1*-mutant plants have normal AtRALF1-induced alkalinization and Ca^2+^ mobilization responses

**Fig 2:**
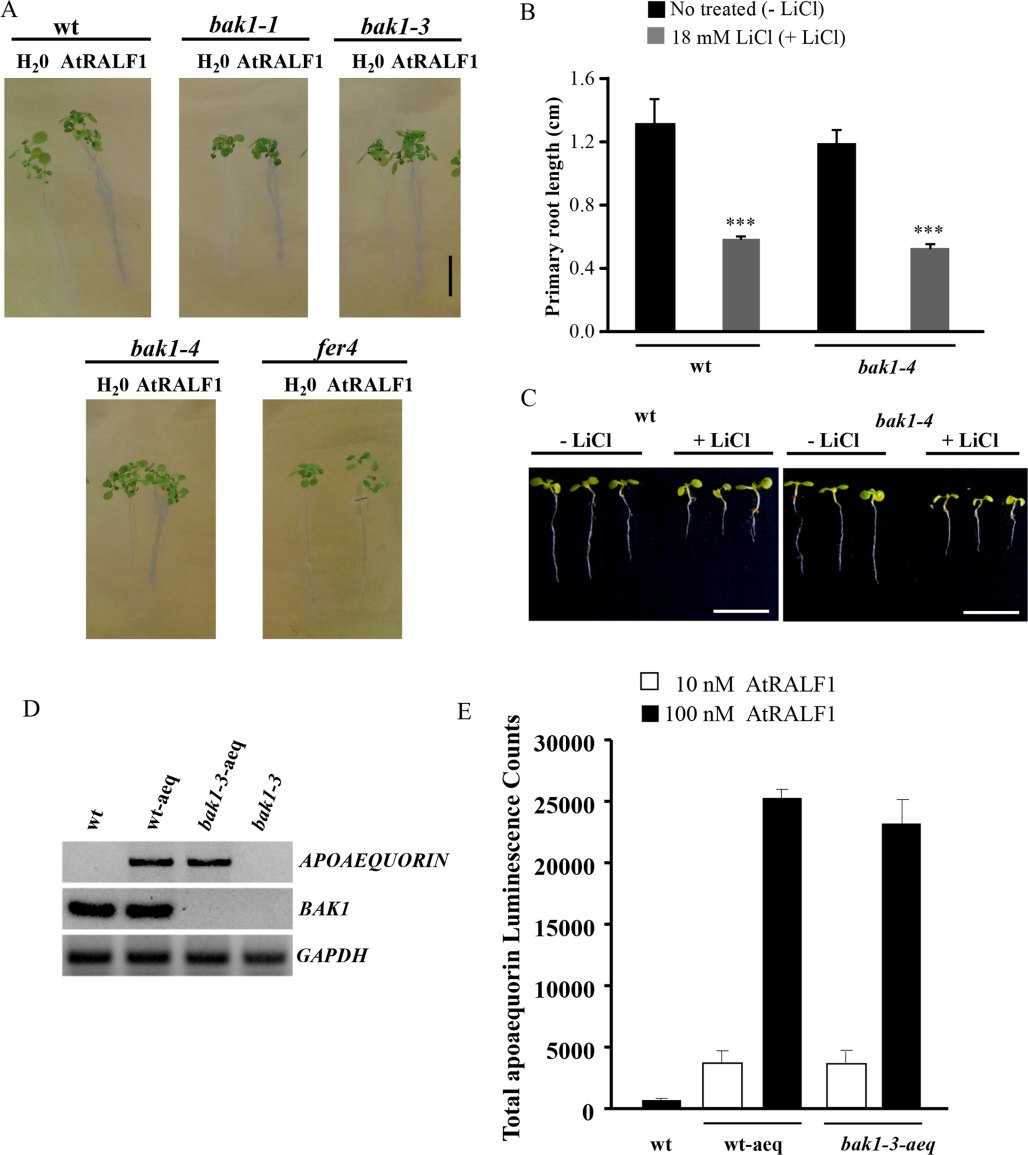
BAK1 is not required for AtRALF1-induced alkalinization and Ca^2+^ mobilization responses. (A) Gel containing the pH-sensitive indicator bromocresol purple. Seven-day-old *Arabidopsis* seedlings were transferred to the gel indicator and treated with 10 μM AtRALF1. Images were captured 30 min after transfer. Scale bar, 1 cm. (B) *Arabidopsis* seedlings (3 days old) were treated for 4 days with 18 mM LiCl. The values are the mean ± SD of at least 20 seedlings. Triple asterisks indicate P < 0.01 (Student’s t-test); ns, not significant. (C) Images of representative individuals. Seedlings were arranged on plates after the treatment for imaging. Scale bar, 1 cm. (D) Expression analysis of *apoaequorin* and *BAK1* genes in wild-type (wt), p35S:APOAEQUORIN (wt-aeq), p35S:APOAEQUORIN/*bak1-3* (*bak1-3*-aeq) and *bak1-3* plants. *GAPDH* expression was used as an internal control. (E) Effects of 10 and 100 nM AtRALF1 on cytosolic Ca^2+^ mobilization in *bak1-3*-aeq transgenic plants. A total of 20 time-points were measured and summed. The results are presented as the mean ± SD of 10 measurements.

AtRALF1 induces a rapid alkalinization of the extracellular media of cell suspensions and a rapid increase in cytoplasmic Ca^2+^ from extra- and intracellular stores [1,11]. *Arabidopsis* growth medium containing the pH indicator bromocresol purple is effective in showing the alkalinization and acidification around the rhizosphere of RALF-overexpressing and wild-type seedlings, respectively [4]. Using a similar method, we showed that wild-type and *bak1* seedlings developed a purple coloration around the rhizosphere upon AtRALF1 treatment, indicating that both genotypes respond to the peptide, causing alkalinization around the roots (Fig 2A). Treatment of the roots with water did not alter the color of the medium. We also included *fer4* mutant for comparison. It has been shown that AtRALF1 perception leads to the phosphorylation of the plasma membrane H^+^-ATPase AHA2, decreasing its activity, and that in the AtRALF1 receptor mutant *fer4*, AHA2 is more active than in the wild-type [10]. The more active AHA2 allows *fer4* plants to acidify the extracellular medium faster than wild-type plants, and the faster acidification confers hypersensitivity to LiCl treatment. If *bak1* mutants have an unaltered AtRALF1-induced alkalinization response, they should not exhibit faster acidification of the extracellular medium, and, consequently, they should respond to LiCl treatment in a manner similar to wild-type seedlings. The exposure of *bak1-4* mutants to LiCl treatment demonstrated that the inhibition of primary root growth in *bak1-4* seedlings treated with LiCl was similar to the inhibition observed in wild-type plants (Fig 2B). Representative images of seedlings after LiCl treatment are shown (Fig 2C). To verify the mobilization of Ca^2+^, we introduced the luminescent Ca^2+^ sensor protein apoaequorin into the *bak1-3-*mutant background, hybridizing the mutant with plants harboring a single insertion of a transgene encoding the apoaequorin protein driven by the cauliflower mosaic virus 35S promoter [11] and selected for the absence of BAK1 gene expression and the presence of the apoaequorin gene. A line homozygous for the apoaequorin transgene and the T-DNA insertion in the *BAK1* gene was selected (Fig 2D). When *bak1-3* plants containing the cytosolic Ca^2+^ indicator were exposed to 10 and 100 nM AtRALF1, they showed a cytosolic calcium increase similar to plants expressing the apoaequorin gene in a wild-type background (Fig 2E). Together, the experiments indicate that BAK1 is not required for the AtRALF1-induced alkalinization of the extracellular media or for the increase in cytoplasmic Ca^2+^ caused by AtRALF1.

### The semi-dwarf phenotype of plants overexpressing AtRALF1 is dependent on BAK1

**Fig 3:**
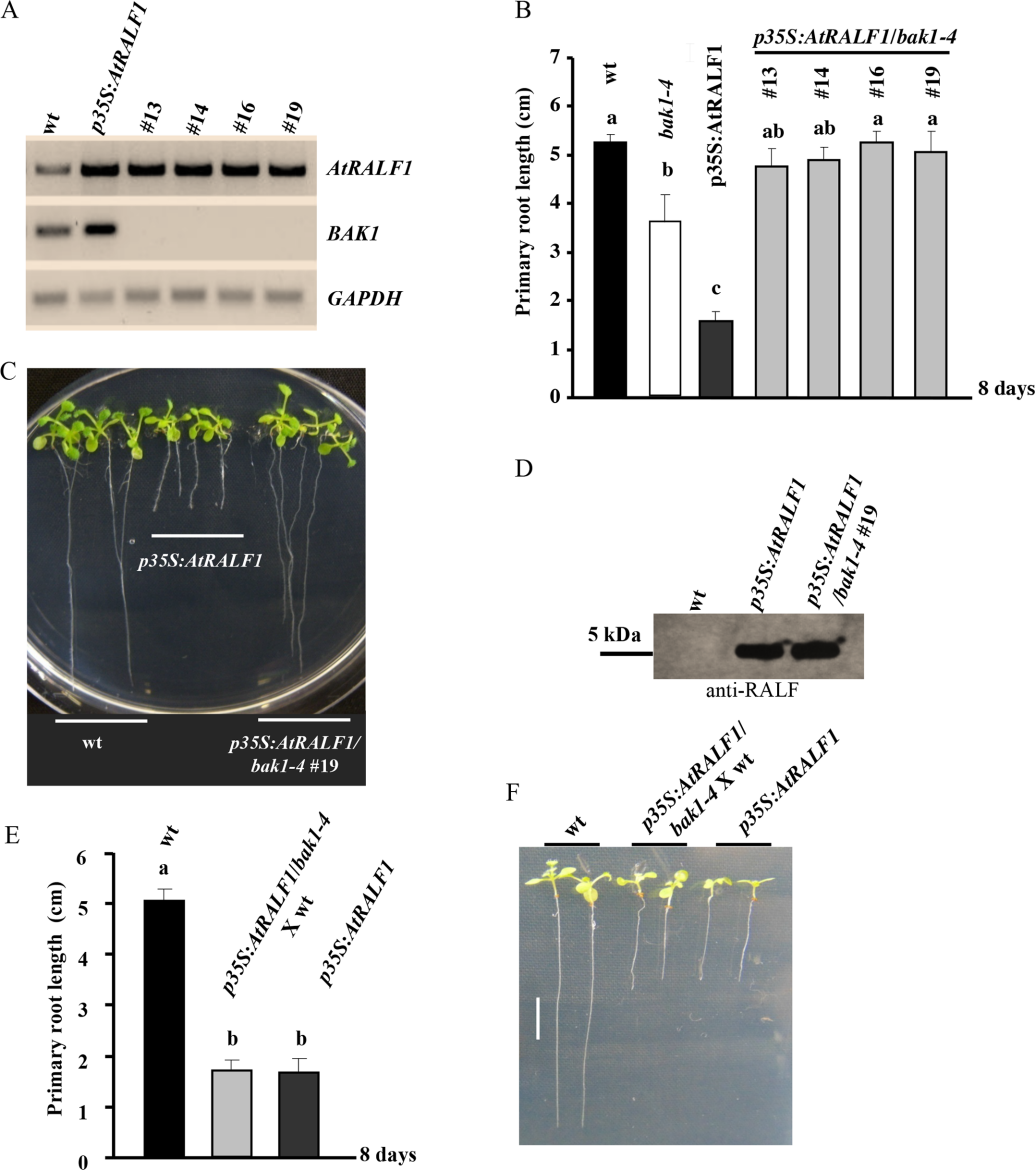
The semi-dwarf phenotype of plants overexpressing the *AtRALF1* gene is BAK1-dependent. (A) Expression analysis of the *AtRALF1* and *BAK1* genes in 8-day-old plants: wt, *p35S*:*AtRALF1* and *p35S*:*AtRALF1/bak1-4* lines #13, 14, 16 and 19. *GAPDH* expression was used as an internal control. (B) *Arabidopsis* seedlings were grown for 8 days in vertical plates for primary root growth measurements. The values are the mean ± SD of 10 plants. The means with the same letter are not significantly different from each other (Tukey’s test, P ≤ 0.01). (C) Representative images of 8-day-old seedlings (wt, *p35S*:*AtRALF1* and *p35S*:*AtRALF1/bak1-4* #19). Scale bars, 1 cm. (D) Blot of protein extracts from *Arabidopsis* seedlings using an anti-RALF antibody. Extracts were separated by reversed-phase chromatography. (E) *Arabidopsis* seedlings were grown for 8 days in vertical plates for primary root growth measurements. The values are the mean ± SD of 10 plants. The means with the same letter are not significantly different from each other (Tukey’s test, P ≤ 0.01). (F) Representative images of 8-day-old seedlings: wt, *p35S*:*AtRALF1/bak1-4* X wt progeny and *p35S*:*AtRALF1*. Seedlings were arranged on plates after the treatment for imaging. Scale bars, 1 cm.

Plants expressing the AtRALF1 gene under the control of the strong viral promoter 35S exhibit a semi-dwarf phenotype with reduced root growth [3]. We hypothesized that if BAK1 is an absolute requirement for AtRALF1-induced root growth inhibition, plants overexpressing AtRALF1 and lacking the *BAK1* gene would exhibit a normal wild-type phenotype. To test this hypothesis, the transgene *p35S*:*AtRALF1* was introduced into the *bak1-*mutant background by crossing semi-dwarf *p35S*:*AtRALF1* plants with *bak1-4* T-DNA-mutant plants. Four homozygous lines for the *p35S*:*AtRALF1* transgene and for the T-DNA insertion in the *BAK1* gene were selected and evaluated. The selected *p35S*:*AtRALF1/bak1-4* lines #13, #14, #16 and #19 showed no detectable *BAK1* expression, and their levels of *AtRALF1* mRNA were similar to the levels found in *p35S*:*AtRALF1* plants (Fig 3A). The primary root length of the four *p35S*:*AtRALF1/bak1-4* homozygous lines, evaluated after 8 days growing on vertical plates, was comparable to that of wild-type plants (Fig 3B). Representative images of plants from the p35S:AtRALF1/bak1-4 line #19 are shown (Fig 3C). The AtRALF1 peptide accumulation in the *p35S*:*AtRALF1/bak1-4* line #19 was similar to the parent line *p35S*:*AtRALF1* (Fig 3D), and when we reintroduced the *BAK1* gene into the *p35S*:*AtRALF1/bak1-4* by backcrossing the hybrid line with the wild-type parental line, the primary root length of the resulting plants was similar to the AtRALF1-overexpressing plants (Fig 3E). Representative images of plants descended from the backcross between *p35S*:*AtRALF1/bak1-4* and wild-type plants are shown (Fig 3F). The lack of the characteristic short-root phenotype in plants constitutively expressing *AtRALF1* in the *bak1* background shows that in plants, the presence of the *BAK1* gene is also necessary for the AtRALF1-induced inhibition of root growth.

### The *BAK1* gene is induced by AtRALF1 and required for the up-regulation of AtRALF1-inducible genes

**Fig 4:**
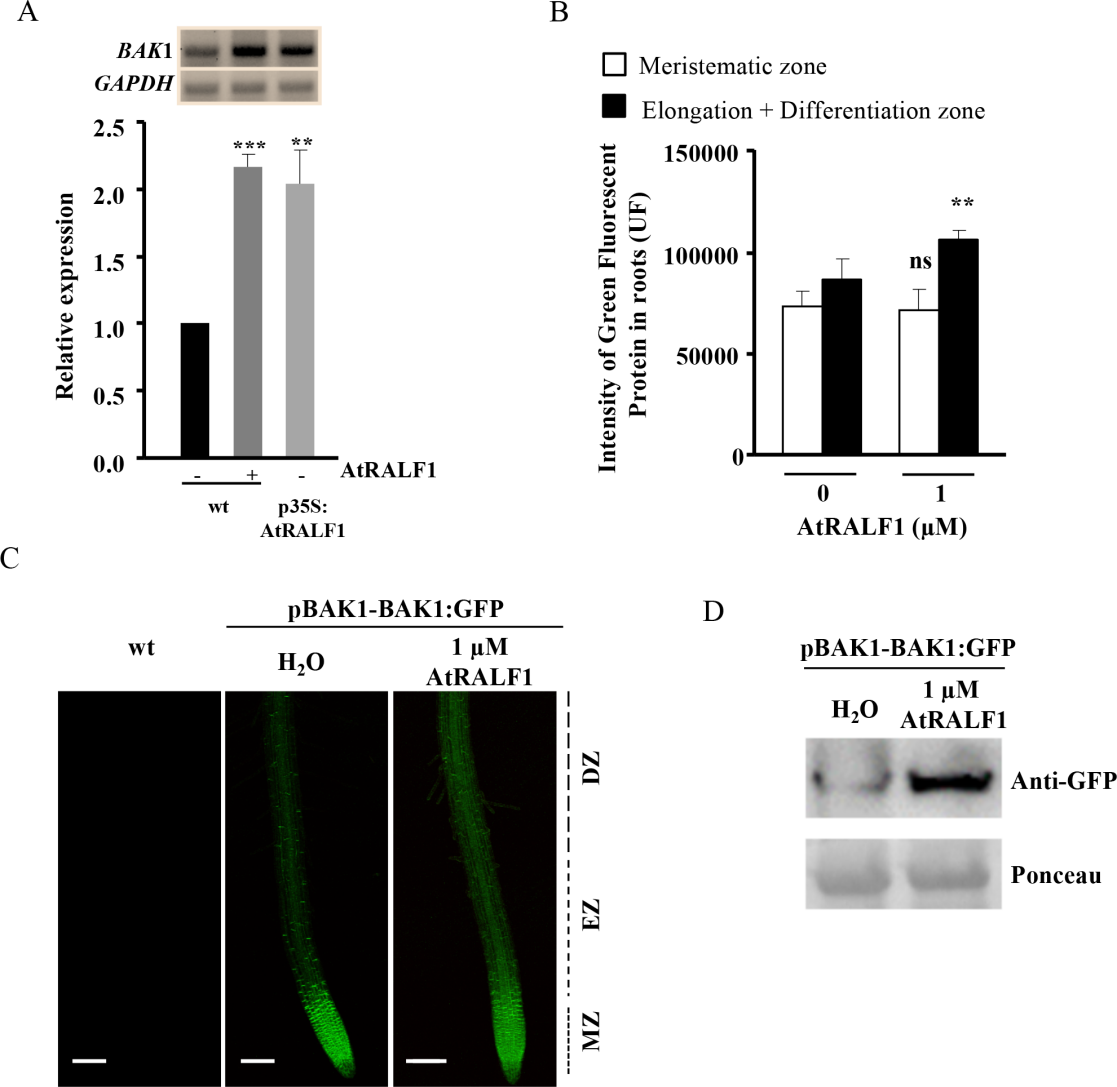
The *BAK1* gene is induced by AtRALF1. (A) BAK1 gene expression in the roots of *Arabidopsis* seedlings (10-days-old) treated with AtRALF1. Root tissues were collected 1 h after treatment. Semi-quantitative RT-PCR and qRT-PCR were performed using total RNA extracted from the roots of untreated (-) or treated (+) plants. *GAPDH* expression was used as an internal control. (B) Quantification of GFP fluorescence intensity. The values are the mean ± SD of 20 seedlings. Student’s t test was used to compare the values obtained from treated and untreated seedlings. Double asterisk indicates P < 0.05 (Student’s t test); ns, not significant. (C) Representative confocal images of pBAK1:BAK1-GFP seedlings (4 days old) treated with 1 μM AtRALF1. Images were captured 4 h after treatment and were obtained by scanning 10 slices over a distance of 7 μm. MZ, meristematic zone; EZ, elongation zone; DZ, Differentiation zone. Bars, 100 μm. (D) pBAK1:BAK1-GFP plants were treated with water or 1 μM AtRALF1. After treatment, protein extracts from pBAK1:BAK1-GFP roots were subjected to immunoblot analysis with anti-GFP antibody. Ponceau was used to show equal loading of protein.

In our search for plants lacking *BAK1* gene expression and overexpressing AtRALF1, we found that the *BAK1* gene was up-regulated in *p35S*:*AtRALF1* plants (Fig 3A). This observation prompted us to investigate whether the *BAK1* gene was an AtRALF1-inducible gene. Quantitative and semi-quantitative RT-PCR analyses showed that the treatment of *Arabidopsis* seedlings with 1 μM AtRALF1 for 1 h induced *BAK1* gene expression in the roots up to levels found in untreated *p35S*:*AtRALF1* roots (Fig 4A). In another experiment, we used pBAK1:BAK1-GFP transgenic plants treated with AtRALF1 to evaluate the responsiveness of the *BAK1* gene promoter to the peptide. The treatment of pBAK1:BAK1-GFP plants with 1 μM AtRALF1 resulted in a quantitative increase in fluorescent signal in the roots (Fig 4B). Because pBAK1:BAK1-GFP untreated plants showed a strong GFP signal in the root meristematic zone, the quantification was performed separately for the meristematic and elongation plus differentiation root zones. Representative images of wild-type, untreated and AtRALF1-treated *Arabidopsis* roots are shown (Fig 4C). BAK1 protein accumulation was also verified by protein blot after *Arabidopsis* seedlings were treated with AtRALF1 peptide (Fig 4D).

**Fig 5:**
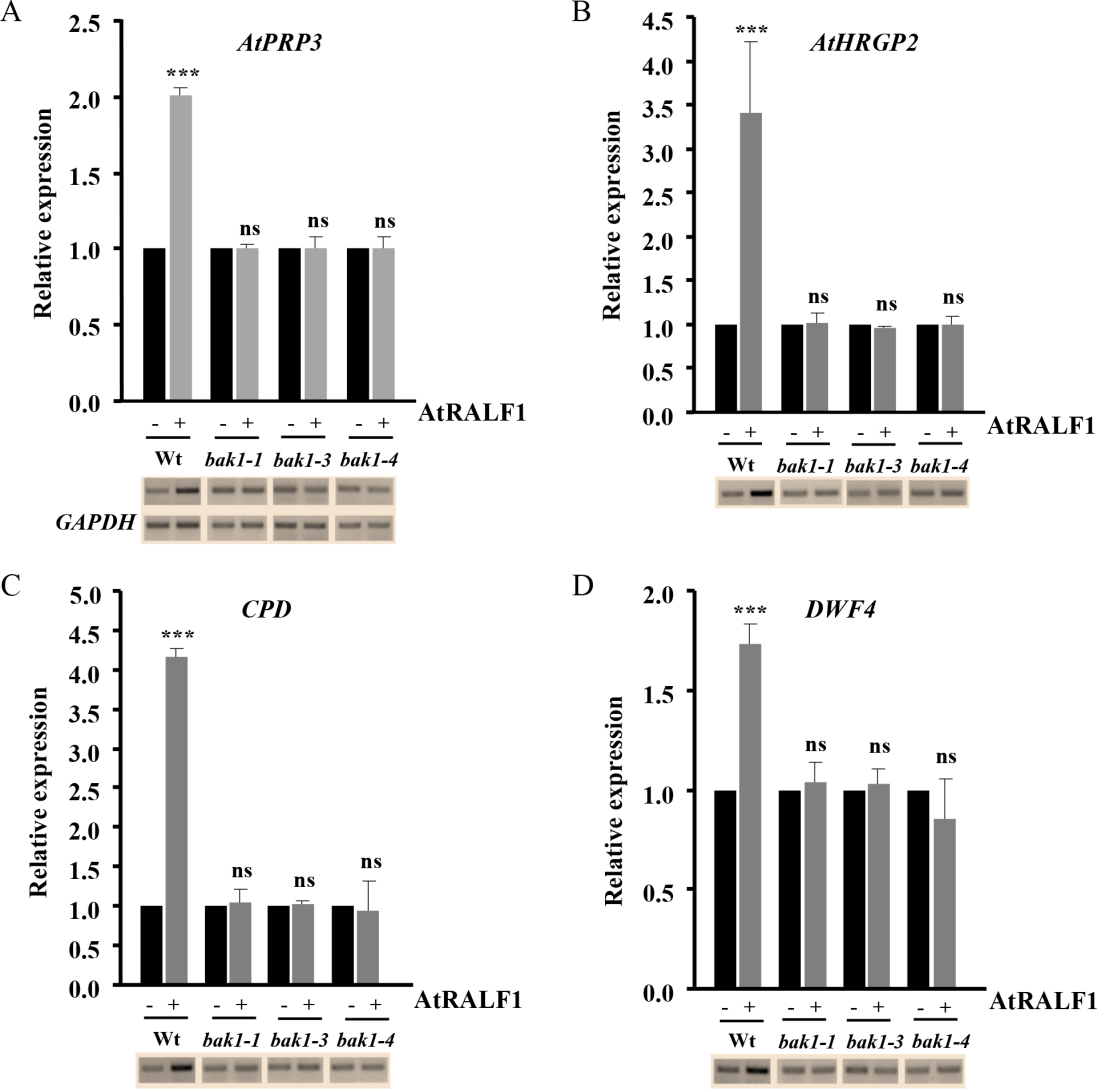
The AtRALF1-inducible genes are not induced in *bak1* mutants. Expression analysis of AtRALF1-inducible genes (A) *AtPRP3*, (B) *AtHRGP2*, (C) *CPD* and (D) *DWF4* in the roots of *Arabidopsis* seedlings (10-days-old) treated for 1 h with 1 μM AtRALF1. Semi-quantitative RT-PCR and qRT-PCR were performed using total RNA extracted from the roots of untreated (-) or treated (+) plants. GAPDH expression was used as a control. *AtPRP3*, PROLINE-RICH PROTEIN3 (At3g62680); *AtHRGP2*, HYDROXYPROLINE-RICH GLYCOPROTEIN2 (At5g19800); *CPD*, CONSTITUTIVE PHOTOMORPHISM AND DWARFISM (At5g05690). *DWF4*, DWARF4 (At3g50660). Triple asterisks indicate P < 0.01 (Student’s t test); ns, not significant.

Exposing *Arabidopsis* seedlings to AtRALF1 peptide induces, in a dose-dependent manner, several genes involved in cell wall rearrangement in their roots, including the proline-rich protein *AtPRP3* and the hydroxyproline-rich glycoprotein *AtHRGP2* [9]. AtRALF1 also induces two cytochrome P450 monooxygenase genes related to the BR biosynthetic pathway: *Constitutive Photomorphism and Dwarfism* (*CPD*) and *DWARF4* (*DWF4*). We exposed the *bak1* mutants to the same concentration of AtRALF1 and evaluated the capacity to induce the *AtPRP3*, *AtHRGP2*, *CPD* and *DWF4* genes. None of the AtRALF1-inducible genes were up-regulated in the *bak1-1*, *bak1-3* and *bak1-4* T-DNA insertion mutants after one hour of AtRALF1 treatment (Fig 5A–5D). Wild-type seedlings treated in the same way showed increased expression of the AtRALF1-inducible genes, ranging from 1.7- to 4.2-fold. Our results using *bak1* mutants demonstrated that the signaling pathway leading to the induction of AtRALF1-responsive genes is not functional in mutants lacking the BAK1 protein.

### AtRALF1 physically interacts with BAK1

**Fig 6:**
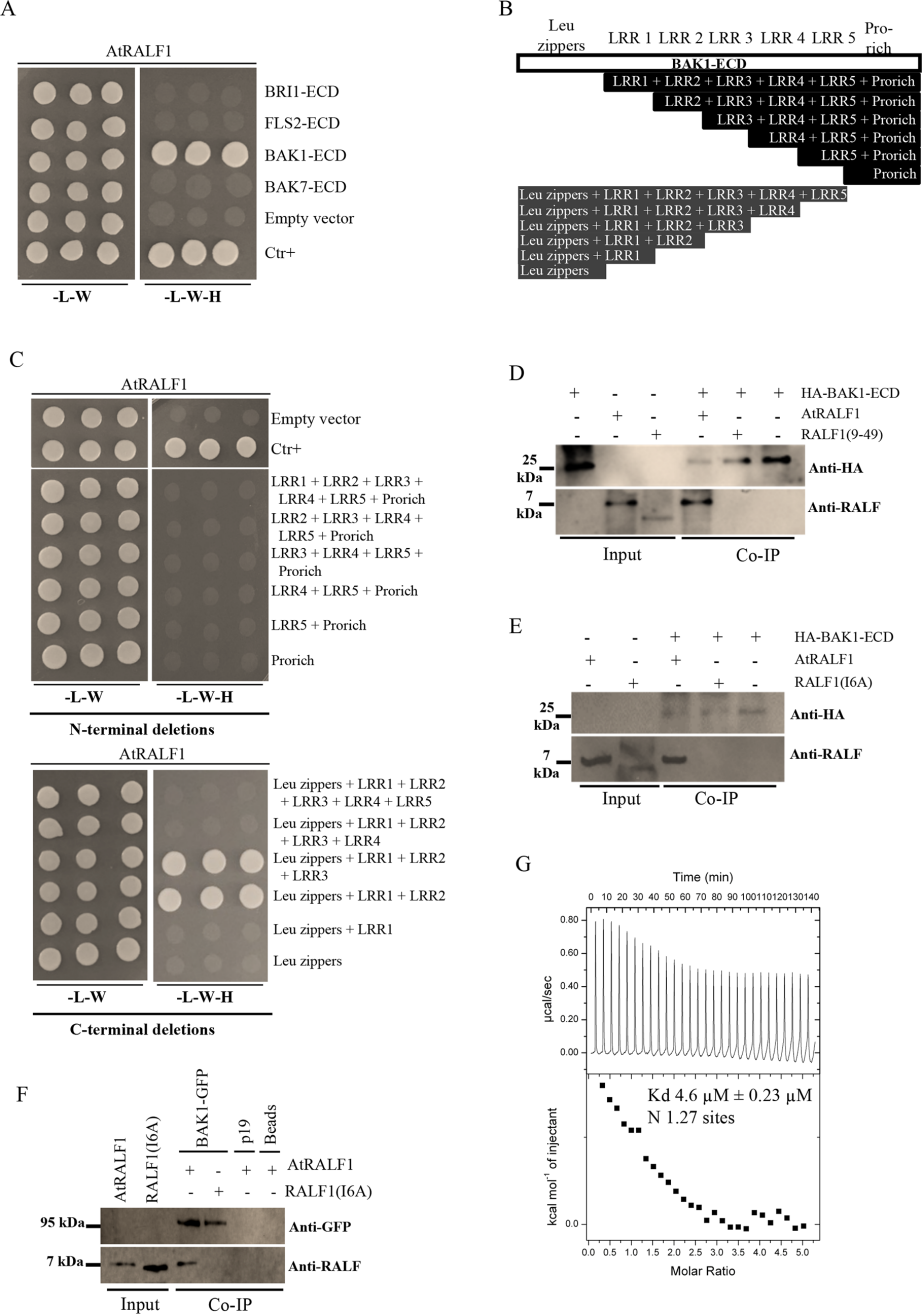
AtRALF1 interacts physically with BAK1 protein. (A) The active AtRALF1 peptide interacts with the extracellular domain (ECD) of BAK1 (BAK1-ECD) in yeast. The AtRALF1 protein fused to the GAL4 DNA-binding domain (AtRALF1-pGBKT7) was used as bait to test its interaction with the extracellular domain of the proteins fused to the GAL4 activation domain (pGADT7): BAK1 (BAK1-ECD), BAK7 (BAK7-ECD), BRI1 (BRI1-ECD) and FLS2 (FLS2-ECD). (B) Blueprint showing the truncated proteins from the extracellular domain of BAK1 tested in the two-hybrid assay. The extracellular domain of BAK1 comprises leucine zippers (LZ), 5 leucine rich-regions (LRRs) and a proline-rich region (pro-rich). (C) AtRALF1-pGBKT7 was used as bait to test its interaction with truncated proteins fused to the GAL4 activation domain (pGADT7). The transformed yeast cells in (A) and (C) were selected on a synthetic complete medium lacking leucine, tryptophan and histidine to test interactions. (D, E) Co-immunoprecipitation (Co-IP) *in vitro* between the extracellular domain of BAK1 (HA-BAK1-ECD) and AtRALF1 recombinant purified proteins. HA-BAK1-ECD was detected with an anti-HA antibody. AtRALF1 was detected with an anti-RALF antibody. (F) Recombinant AtRALF1 is co-immunoprecipitated with the BAK1-GFP protein expressed in tobacco. BAK1-GFP was detected with an anti-GFP antibody. The synthetic and inactive peptides RALF1(9–49) and RALF1(I6A) did not co-immunoprecipitate with BAK1. (G) Isothermal titration calorimetry (ITC) of the extracellular domain of BAK1 (BAK1-ECD) vs. AtRALF1 in ITC buffer (15 mM HEPES, pH 6.8). ITC experiments were simulated using 28 injections of BAK1-ED (0.7 mM, vol 10 μL) on AtRALF1 (0.03 mM vol cell = 1.4 mL) at 25°C. K_d_ = dissociation constant; N = number of binding sites.

To investigate the physical interaction between AtRALF1 and BAK1, we first confirmed that both proteins were present in the same root cell type. *In silico* data showed that both genes are expressed in root cells, which we confirmed by analyzing transgenic plants harboring the chimeric genes pAtRALF1:AtRALF1-GFP and pBAK1:BAK1-GFP. The AtRALF1-GFP fusion protein was found in the endodermis of the differentiation zone of the roots of pAtRALF1:AtRALF1-GFP transgenic plants (S10 Fig). Analysis of pBAK1:BAK1-GFP transgenic plants using confocal microscopy showed that BAK1 was expressed in all root cell types, including the endodermis of the differentiation zone (S11 Fig). To investigate the potential interaction between AtRALF1 and BAK1 proteins, we first used a yeast two-hybrid (Y2H) system. The extracellular domain (ECD) of BAK1 (BAK1-ECD) fused to the GAL4 activation domain and the coding region of the AtRALF1 peptide fused to the GAL4 binding domain were co-transformed into yeast and grown in selective medium (Fig 6A). BAK7 (BAK7-ECD), BRI1 (BRI1-ECD) and FLS2 (FLS2-ECD) are all leucine-rich-repeat-containing proteins, and their ECDs were fused to the GAL4 activation domain as controls to test BAK1-ECD specificity. The AtRALF1 construct was able to complement only the BAK1-ECD construct in the selective medium (Fig 6A). We attempted to map the regions of the extracellular domain of BAK1 that would be absolutely required for the interaction with AtRALF1. Therefore, a series of deletions from N-terminal and C-terminal regions of the extracellular domain of BAK1 were generated and transformed into yeast (Fig 6B), and the protein expression was verified by immunoblotting using anti-hemagglutinin (anti-HA) and anti-cMYC epitope antibodies (S12 Fig). Among the 12 truncated proteins, we found only two that interacted with AtRALF1: Leu zippers + LRR1 + LRR2 + LRR3 and Leu zippers + LRR1 + LRR2 (Fig 6C). None of the deletions from N-terminal were able to interact with AtRALF1, indicating that an intact N-terminal is essential for the interaction. We used another AtRALF fused to the binding domain to further test specificity. The coding region of the active AtRALF34 peptide was co-transformed into yeast with the ECDs of the LRR-containing proteins, BAK1, BAK7, BRI1 and FLS2, and no complementation was observed (S13 Fig).

The interaction between AtRALF1 and BAK1-ECD was further confirmed *in vitro* using co-immunoprecipitation assays. Chimeric AtRALF1 and BAK1 proteins containing the His-tag fused to the N-terminus were expressed in *E*. *coli* and purified using affinity chromatography. His-tagged AtRALF1 was obtained as previously described [5]. The His-tagged BAK1-ECD also contains a HA-tag fused to the N-terminus. The HA-BAK1-ECD HPLC profile and mass spectrometer data are shown (S14 Fig). Recombinant AtRALF1 was incubated with HA-BAK1-ECD protein and co-immunoprecipitated using agarose anti-HA beads (Fig 6D and 6E). The inactive peptides RALF1(9–49) and RALF1(I6A) did not co-immunoprecipitate with BAK1, suggesting that the interaction requires a functional AtRALF1. We repeated the immunoprecipitation, incubating AtRALF1 with the microsomal fraction of *Nicotiana benthamiana* leaves transiently expressing the entire BAK1 protein fused to GFP (BAK1-GFP). AtRALF1 co-immunoprecipitated when the anti-GFP antibody was used (Fig 6F). RALF1(I6A) peptide did not co-immunoprecipitate with BAK1-GFP, and AtRALF1 was not co-immunoprecipitated when incubated with either P19 alone or Sepharose beads. Using isothermal titration calorimetry (ITC), we titrated a known concentration of AtRALF1 with small injections of BAK1-ECD (Fig 6G). ITC experiments were used to determine the dissociation constant (K_d_) of the AtRALF1/BAK1 interaction and the number of AtRALF1 binding sites (N). Reverse ITC experiments were performed because AtRALF1 exhibits heat of dissociation (S15A Fig). In three ITC experiments performed the estimated K_d_ were 4.6, 4.9 and 5.8 μM and in all three experiments the number of binding sites was near 1 (1.27, 1.5, 0.5). Fig 6G shows one representative ITC experiment. Heat generated by injection of BAK1-ECD into ITC buffer was subtracted and the heat generated by ITC buffer injected into AtRALF1 was negligible (S15B and S15C Fig). When AtRALF1 was replaced by the inactive RALF1(9–49), ITC experiments detected only the heat generated by BAK1-ECD (S16A–S16C Fig), demonstrating no interaction between the inactive analog and BAK1-ECD. Both the Y2H and co-immunoprecipitation experiments demonstrate a physical interaction between AtRALF1 and BAK1 that, together with the gene coexpression data, suggests that AtRALF1 and BAK1 could interact *in planta* with an affinity that our ITC data demonstrate is compatible with a ligand/co-receptor interaction.

### AtRALF1 labeled with chemiluminescent acridinium (acriAtRALF1) shows decreased specific binding in *bak1* mutants

**Fig 7:**
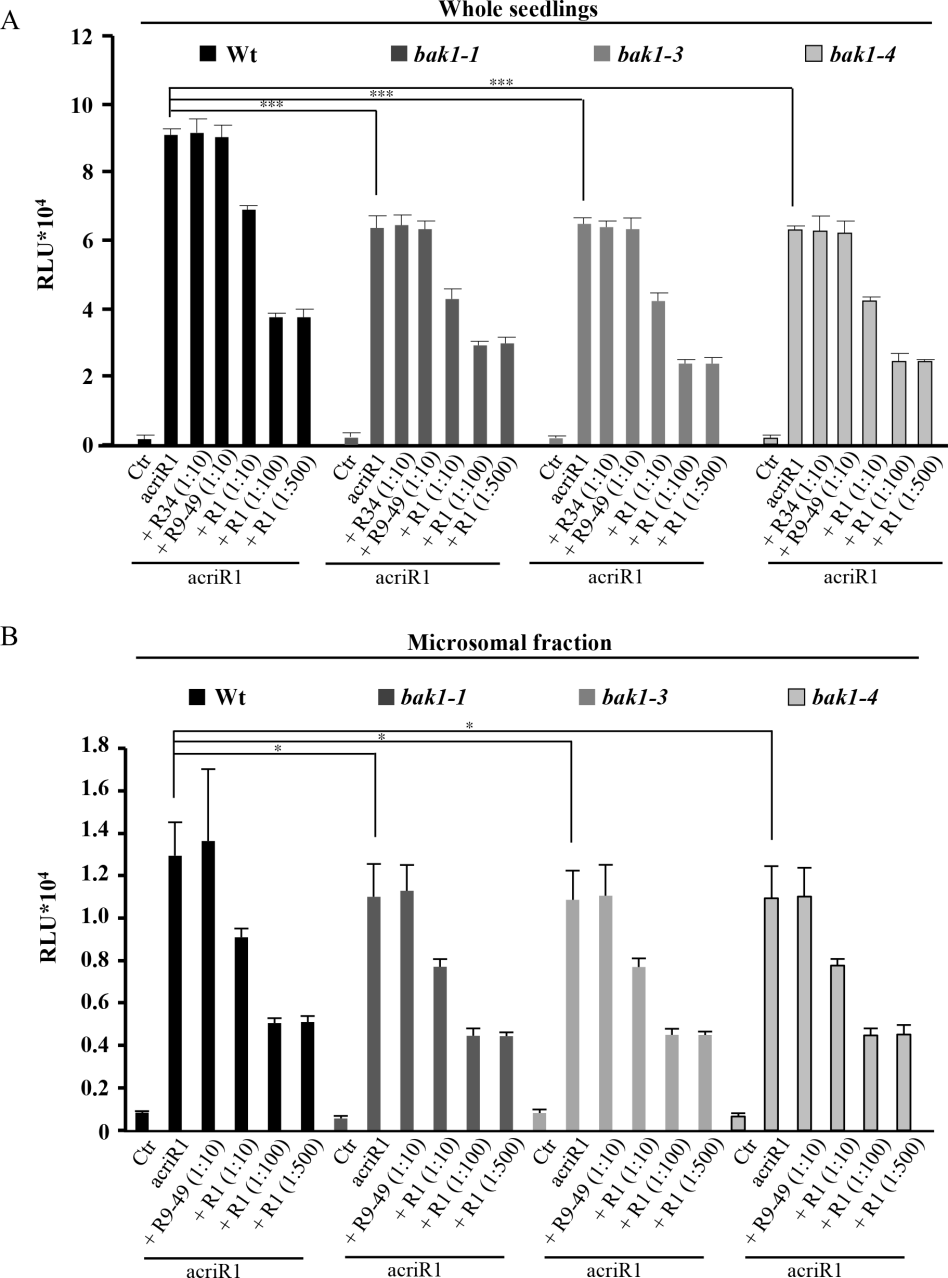
AtRALF1 labeled with chemiluminescent acridinium (acriAtRALF1) shows decreased specific binding in *bak1* mutants. (A) Whole seedlings (5 days old) were treated for 15 min with acriAtRALF1 (acriR1), acriR1 and an excess of unlabeled AtRALF34 (R34), RALF1(9–49) [(R-9-49)], or AtRALF1 (R1), as indicated. The values are the mean ± SD of two measurements (5 seedlings each). (B) Microsomal fractions from wild-type and *bak1* mutants were incubated for 15 min with acriAtRALF1 (acriR1), with acriR1 and an excess of unlabeled RALF1(9–49) [(R-9-49)], or with AtRALF1 (R1), as indicated. The values are the mean ± SD of two measurements. The proportions of labeled: unlabeled proteins are shown in parentheses. Single and triple asterisks indicate P < 0.1 and P < 0.01 (Student’s t test).

Acridinium-labeled peptides have been proposed to be sensitive probes to detect specific protein-binding sites in plants [32]. Acridinium esters covalently bind to proteins and produce chemiluminescence in the presence of hydrogen peroxide, and thus, they can be used as an efficient tool to detect labeled proteins. To investigate the importance of BAK1 in AtRALF1 binding, we produced and purified an acridinium-labeled AtRALF1 (acriAtRALF1) peptide. To verify the biological activity of acriAtRALF1, we performed an apoaequorin/Ca^2+^ assay, which showed that both peptides, unlabeled AtRALF1 and acriAtRALF1, induced an increase in cytoplasmic Ca^2+^ (S17A Fig). Intact wild-type seedlings incubated with acriAtRALF1 retained a detectable amount of acriAtRALF1 that could not be competed out by a 10-fold excess of unlabeled AtRALF34 or inactive AtRALF1(9–49) (Fig 7A). When a 10-fold excess of unlabeled AtRALF1 was added, a 24% reduction was observed, indicating that the retained luminescence was due to the specific binding of the acriAtRALF1 and that this binding could be competed out by the unlabeled peptide. Binding was reduced even further, up to 59%, when a 100-fold excess of AtRALF1 was added. No further reduction was observed, even when a 500-fold excess was used. To avoid background signal from the aerial parts of the seedling, we confirmed that the roots were the only source of the chemiluminescent signal (S17B Fig). Compared with that of the wild-type seedlings, the total acriAtRALF1 binding was reduced by 30, 29 and 31% in *bak1-1*, *bak1-3* and *bak1-4* seedlings, respectively (Fig 7A). Total acriAtRALF1 binding was reduced by approximately 48% in *fer4* mutants (S17C Fig). A similar result in *fer4* mutants was previously reported using RALF labeled with ^125^I [10]. Similar to wild-type, incubation with a 10-fold excess of unlabeled AtRALF34 and AtRALF1(9–49) did not reduce the binding in the mutant backgrounds. When a 10-fold excess of AtRALF1 was used, reductions in binding of approximately 32, 35 and 33% were detected in the *bak1-1*, *bak1-3* and *bak1-4* mutants, respectively. A 100- or 500-fold excess of AtRALF1 reduced acriAtRALF1 binding to 54, 63 and 61% in *bak1-1*, *bak1-3* and *bak1-4* mutants, respectively. As a control, *bak7* mutants were incubated with acriAtRALF1 in the same conditions, and no differences in the reduction of total binding between wild-type and *bak7* mutants were observed (S18A Fig). As an additional control, we produced acriAtRALF34, the same isoform that was able to inhibit the root growth of the *bak1* mutants. No differences in reduction of total binding were observed when the wild-type and *bak1* mutants were compared using acriAtRALF34 in binding experiments (S18B Fig). Incubation with a 10-fold excess of unlabeled AtRALF1 did not alter the total binding. We next evaluated the acriAtRALF1 binding in microsomal fractions from the roots of wild-type, *bak1-1*, *bak1-3* and *bak1-4* seedlings. The total acriAtRALF1 binding in the microsomal fractions of the *bak1* mutants tested was reduced by approximately 15% compared with that of the wild-type (Fig 7B). The binding of acriAtRALF1 in the microsomal fractions was also specific and saturable. Incubation with a 10-fold excess of unlabeled AtRALF1 reduced the binding to approximately 30% of the total in the microsomal fractions of wild-type and *bak1* plants. A 100- or 500-fold excess of AtRALF1 reduced acriAtRALF1 binding to approximately 60% of the total binding in the microsomal fractions of wild-type and *bak1* plants. These binding experiments suggest that BAK1 is responsible for approximately 30% of AtRALF1 binding in the intact roots of *Arabidopsis* and approximately 15% of AtRALF1 binding in the microsomal fractions derived from the roots of *Arabidopsis*. The difference in the reduction of specific binding found between experiments performed with intact seedlings and with microsomal fractions suggests the existence of an apoplastic component that might assist in AtRALF1 binding.

### BAK1 phosphorylation increases in roots of *Arabidopsis* after AtRALF1 treatment

**Fig 8:**
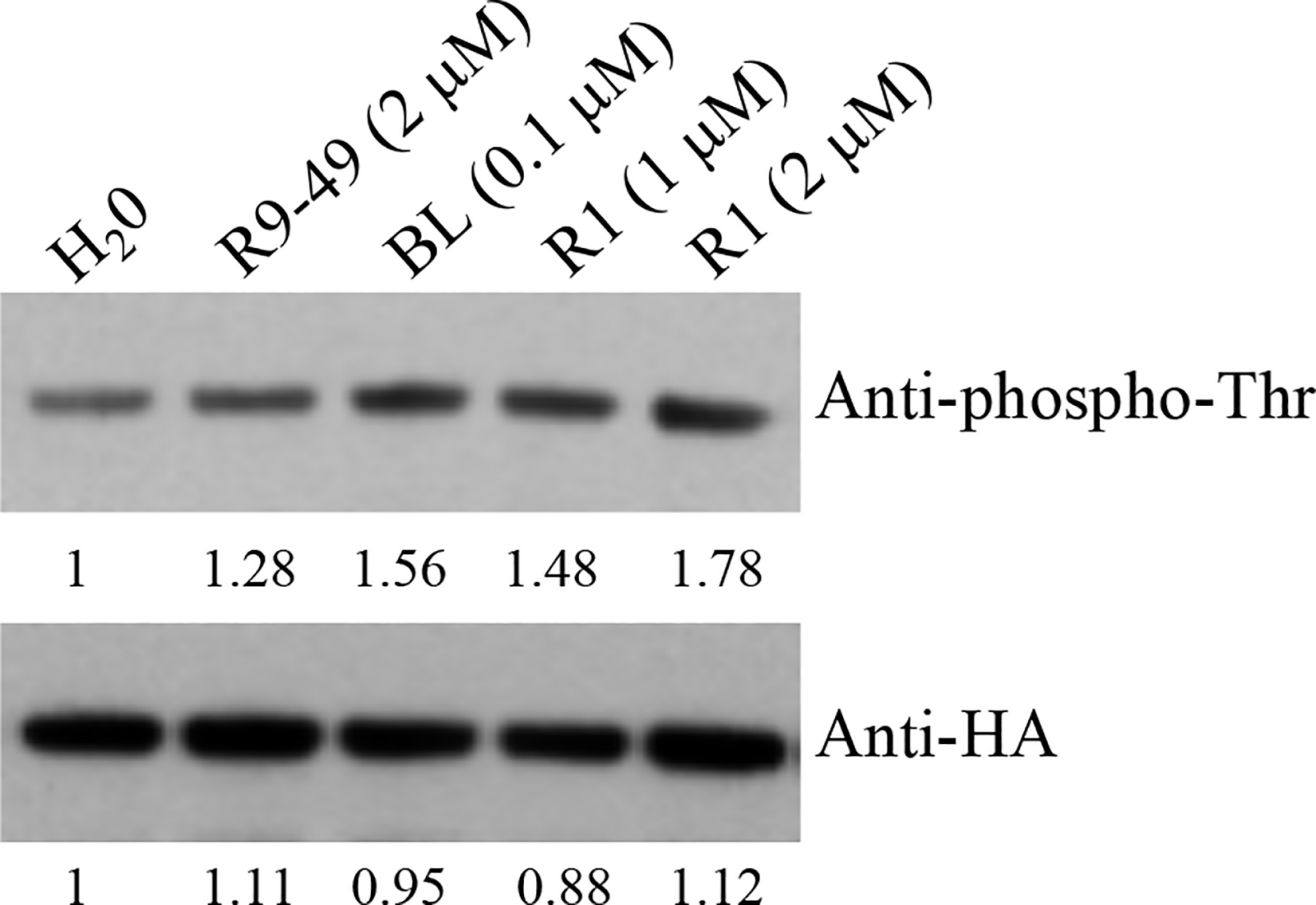
BAK1 phosphorylation increases in roots of *Arabidopsis* after AtRALF1 treatment in a dose-dependent manner. pBAK1:BAK1-6xHA plants were treated with water (H_2_0), 2 μM RALF1(9–49) [R9-49], 0.1 μM BL, 1 and 2 μM AtRALF1 for 20 min. After treatment, the microsomal fraction was isolated from roots. BAK1 protein was immunoprecipitated with anti-HA beads and subjected to immunoblot analysis with anti-phospho-Thr and anti-HA antibodies. The numbers indicate the relative intensity of the bands.

BAK1 has an active cytoplasmic kinase domain that can phosphorylate itself or be phosphorylated by other plasma membrane proteins such as BRI1 [21]. Phosphorylation of serine, threonine and tyrosine residues in the BAK1 kinase domain has been reported [21,33]. To investigate whether AtRALF1 would also change the level of phosphorylation of BAK1, seedlings were exposed to the peptide, and protein blots containing BAK1-HA-flagged proteins immunoprecipitated from the protein extracts of pBAK1:BAK1-HA roots were probed with an anti-phosphothreonine antibody. AtRALF1 treatment caused an increase in BAK1 phosphorylation in the threonine residues similar to the increase resulting from the treatment of the seedlings with BL and showed a dose-dependent response up to 2 μM. High concentrations such as 10 μM did not result in further increases in phosphorylation (Fig 8 and S19 Fig). BAK1 phosphorylation after AtRALF1 treatment was also evaluated using anti-phosphoserine antibody and no changes were detected (S20 Fig). LRR-RK proteins such as BAK1 convey extracellular messages through the activation and phosphorylation of their cytoplasmic kinase domains. The observation that AtRALF1 induces an increase in BAK1 phosphorylation suggests that the binding of AtRALF1 to BAK1 is functional.

## Discussion

The AtRALF1 peptide negatively regulates cell expansion and has been shown to counteract the effects of BR on *Arabidopsis* roots [9]. In a search for putative genetic components that might be shared by both the AtRALF1 and BR signaling pathways, we tested mutants lacking different components of the BR signaling pathway in root growth assays with inhibitory concentrations of the AtRALF1 peptide. Three BAK1 T-DNA insertion mutants, *bak1-1*, *bak1-3* and *bak1-4*, showed insensitivity to the AtRALF1 peptide (Fig 1C and S1 Fig). A previous study in which 18 receptor-like proteins were tested in root growth inhibition assays in the presence of 1 μM AtRALF1 showed that the roots of the *bak1-4* mutant were inhibited by the peptide [10]. In our study, *bak1-1*, *bak1-3* and *bak1-4* were confirmed for the insertion of the T-DNA and for the lack of expression of BAK1 (S3 Fig). We used two different experimental setups, liquid and semi-solid media (vertical plates), and all of them showed insensitivity despite the use of high concentrations of the peptide. When the lengths of the endodermic cells of the primary roots of wild-type and *bak1* mutants were measured under the microscope, the AtRALF1-treated *bak1* was not different from the water-treated wild-type control (Fig 1D). That result was somewhat unexpected, because the roots of *bak1* mutants are shorter than those of the wild-type, and our earlier experiments had shown that BAK1 was required for the root growth inhibition activity caused by AtRALF1 (Fig 1A–1C) [21]. In accordance with these premises, the root cells of the *bak1* mutants should be shorter than those of the wild-type, or the *bak1* mutants should exhibit fewer and longer cells. BAK1 is a common co-receptor that plays a role in various cell responses related to growth, development and defense against pathogens, which compounds the difficulty of using *bak1*-mutant phenotypes to make inferences regarding its function [21–26]. Moreover, previous studies have indicated that mutants defective in the BR pathway have smaller roots due to a reduced number of cells in the root meristem [34–36].

Two biological activities of RALF peptides, the alkalinization of the extracellular medium and the capacity to inhibit root growth, have been intuitively associated mainly because of the counter effect of alkalinization on the acidic apoplastic pH required to sustain root growth. In fact, a mechanism for such counter effects has been proposed, in which AtRALF1 binds the plasma membrane protein receptor FERONIA, and upon RALF-FERONIA interaction, the plasma membrane H^+^-ATPase is inhibited by phosphorylation, mediating the increase of the extracellular pH and the consequent inhibition of cell expansion [10]. Our evaluation of the AtRALF1-induced alkalinization and Ca^2+^ mobilization of *bak1* mutants suggests that, in contrast to the root inhibitory activity, both alkalinization and Ca^2+^ mobilization are independent of the *BAK1* gene (Fig 2). One possibility that would require further experimental testing is the involvement of BAK1-LIKE proteins such as BKK1, which in some circumstances has been shown to replace BAK1 function [37]. In that case, BKK1 would replace only the ion-flux part of the AtRALF1-induced responses, because we tested BKK1 in the root inhibition assay, and the response was similar to wild-type seedlings (S5 Fig). The BL-induced mobilization of Ca^2+^ has also been shown to be independent of *BAK1* [38].

The *BAK1* gene is up-regulated in AtRALF1-overexpressing plants and induced by AtRALF1 treatment (Fig 4A–4D). It has been previously shown through microarray experiments that the treatment of *Arabidopsis* plants with AtRALF1 causes *BAK1* gene induction by approximately 1.56-fold [10]. Our data are consistent with that previous observation and add a new gene to the list of AtRALF1-inducible genes. BL hormone treatment also promotes *BAK1* gene induction in *Arabidopsis* plants [29]. The fact that AtRALF1 peptide and BL treatment both induce *BAK1* gene expression provides further evidence that *BAK1* is a component shared by both pathways. AtRALF1-inducible genes were not induced in *bak1* mutants exposed to AtRALF1 (Fig 5). Thus, the lack of induction of AtRALF1-inducible genes and the normal alkalinization and Ca^2+^ response of the *bak1* mutants suggest the existence of distinct AtRALF1 response pathways. Distinct pathways involved in BR signaling have also been suggested based on Ca^2+^ response and gene expression [38].

A physical interaction between AtRALF1 and BAK1 was demonstrated both in yeast and *in vitro* (Fig 6). Our attempt to map the BAK1-ECD regions necessary for AtRALF1 binding demonstrated an absolute requirement of an intact N-terminus, and although the deletion of the Pro-rich region and the last Leu-rich-repeat (LRR5) abolished the interaction, our Y2H results showed that the Leu zippers, LRR1, and LRR2 are sufficient to support the AtRALF1/BAK1 interaction. The N-terminal capping domain of LRR proteins has been shown to be well conserved and thus critical for structure and complex formation, and in the case of the BRI1LRR-BL-BAK1LRR complex, the N-terminal residues BAK1^Trp59, Phe60, His61^ are important for ligand recognition [39,40].

Isothermal titration calorimetry has been used to estimate K_d_ values for plant ligand-receptor kinase pairs, IDA/ HAESA and AtRALF23/FERONIA are among them [41,42] and our K_d_ values are consistent with these reported values. Our binding data using the acridinium-labeled AtRALF1 also demonstrate that BAK1 is an additional site for AtRALF1 binding in the plasma membrane of *Arabidopsis* roots (Fig 7). Previous studies have shown the possibility of additional sites for AtRALF1 binding. In cell suspension cultures of tomato, tobacco and alfalfa, the ^125^I-azido-RALF peptide cross-linked to two plasma membrane proteins of 25 kDa and 120 kDa [15]. The alkalinization assay testing the sequential addition of saturating concentrations of different AtRALFs suggests that there is more than one AtRALF1 binding site in *Arabidopsis* cell suspension cultures [5]. Finally, in the mutant of AtRALF1 receptor protein *FERONIA*, *fer4*, the insensitivity to AtRALF1 is only partial: the specific binding of ^125^I-labeled AtRALF1 was reduced by approximately 40% [10], and our own results show a binding reduction with acridinium-labeled AtRALF1 of approximately 48% in *fer4* mutants (S17C Fig). Intact seedlings and microsomal fraction from the different genotypes incubated with acriAtRALF1 retained a detectable amount of acriAtRALF1 that could not be competed out by an excess of non-labeled peptide, indicating that portion of the binding was not specific, as previously shown [10]. Even though, a binding reduction of 30% could still be observed in *bak1*-intact seedlings, in comparison to wild type, suggesting again that BAK1 is an additional AtRALF1 binding site and may be part of a AtRALF1-containing complex. However, as BAK1 contributed to only 15% of the AtRALF1 binding in microsomal fractions, we propose that BAK1 may function assisted by apoplastic yet unidentified factors. Future studies should be performed to address whether FERONIA, AtRALF1 and BAK1 form a complex.

**Fig 9:**
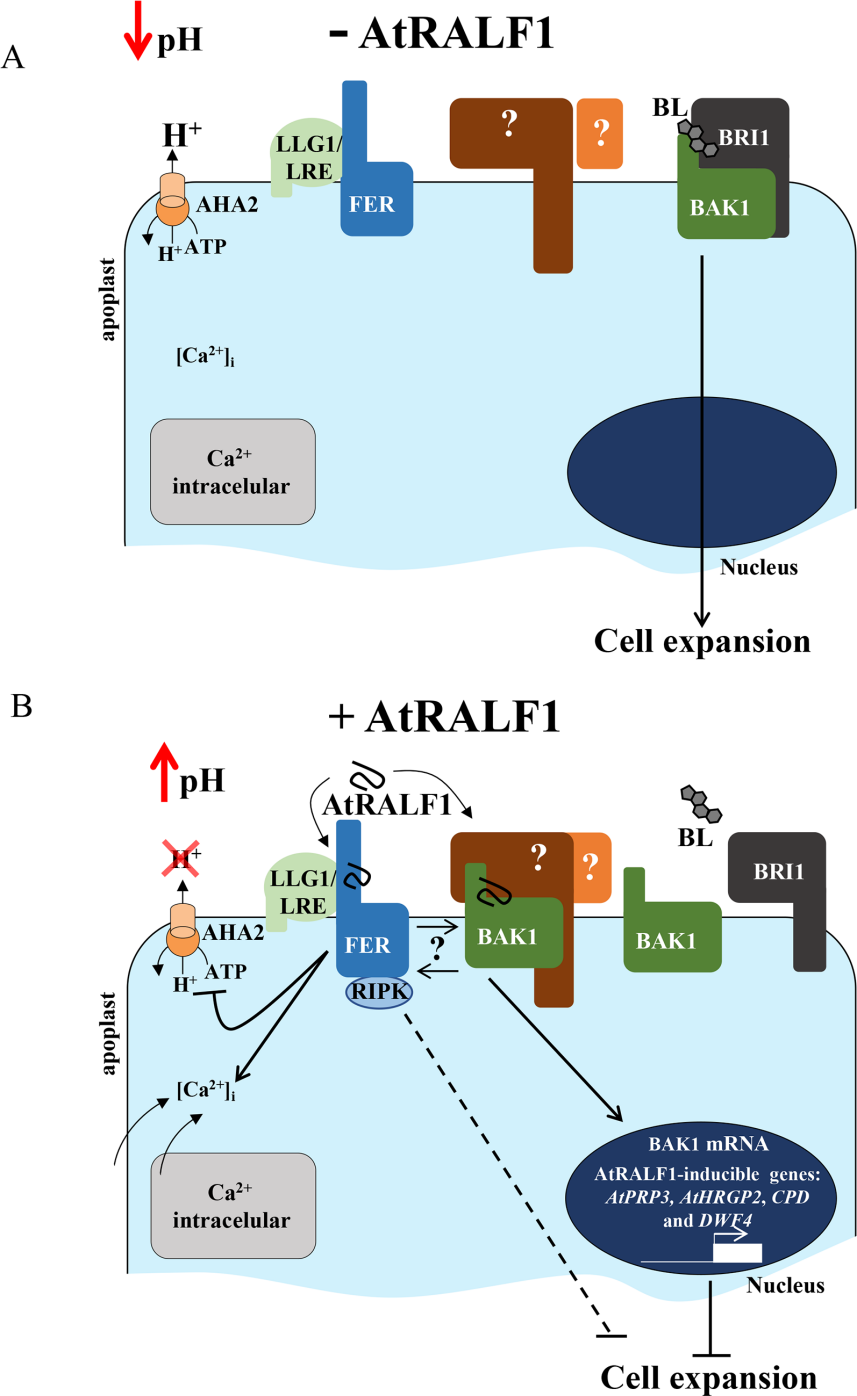
Proposed model for AtRALF1 perception in root cells of Arabidopsis. (A) In the absence of AtRALF1, the BRI1-BL-BAK1 complex is active in the plasma membrane and the cell expands. The apoplast is acidic as the plasma membrane proton pump AHA2 is functional and FERONIA (FER) is in the cell membrane complexed with LRE-like GPI-AP1 (LLG1)/LORELEI (LRE). (B) In the presence of AtRALF1, the peptide binds FER-LLG1/LRE complex, recruits the receptor-like cytoplasmic kinase RIPK and inactivates AHA2, leading to an increase in apoplastic pH. It is proposed that the resulted apoplastic alkalinization dissociates the BRI1-BL-BAK1 complex, allows AtRALF1 to bind BAK1, disrupts BL signaling, activates AtRALF1-inducible genes and, ultimately, inhibits cell expansion. According to our model, AtRALF1/BAK1-dependent responses are downstream of intracellular Ca^2+^ mobilization and apoplastic alkalinization. Both FER and BAK1 may interact, and an apoplastic factor, and at least another receptor is expected to play a role in AtRALF1 perception (question marks). The intersecting line indicates incomplete insensitivity to inhibition of cell expansion caused by AtRALF1; solid lines depict direct actions (with bars at the end = inhibition or arrowed = activation). For more details, see the Discussion.

Based on our findings and previous reports in the literature, a model for AtRALF1 perception that involves BAK1 is proposed (Fig 9A and 9B). It has been shown that the BL-induced BRI1LRR-BAK1LRR complex formation is pH dependent, with acidic values favoring the interaction [40]. In the absence of AtRALF1, the H^+^-ATPase AHA2 is active, the pH of the apoplast is low and brassinolide (BL) forms a complex with BRI1 and BAK1 conveying the message for cell expansion (Fig 9A). Under AtRALF1 physiological concentrations, the peptide alone is not able to dissociate the BRI1LRR-BL-BAK1LRR complex. However, upon binding to FERONIA, with the consequent induction of the alkalinization of the apoplast, the increased pH disassembles the complex, allowing the binding of AtRALF1 to BAK1, precluding the formation of a new BRI1LRR-BL-BAK1LRR complex (Fig 9B). This scenario is consistent with AtRALF1 counteracting the effects of BL and with the binding of AtRALF1 to BAK1 being a requirement for root growth inhibition but playing no role in the alkalinization. Our proposed model explains the partial insensitivity of the *fer4* mutants to the root inhibitory effect of AtRALF1. Low concentrations of AtRALF1 are not able to displace BAK1 in the absence of FER, however, high concentrations of the AtRALF1 peptide would eventually compete with and disassemble the BRI1LRR-BL-BAK1LRR complex without the assistance of alkalinization, overridden the AtRALF1/FER interaction and subsequent AHA2 inhibition. Because the alkalinization induced by AtRALF1 is transient, it would be responsible for the dissociation of the BRI1LRR-BL-BAK1LRR complex upon binding to FER. The dissociation would then allow BAK1 to bind AtRALF1, triggering the up- and down-regulation of cell wall rearrangement genes that ultimately would inhibit cell expansion [9,18]. This mechanism explains the partial insensitivity of the *fer4* mutants and the opposing effects of AtRALF1 and BRs. Our data demonstrate that the BAK1 protein is a newly identified binding site for the AtRALF1 peptide in the roots of *Arabidopsis* seedlings, and may play a role in AtRALF1 signaling as a co-receptor.

## Materials and methods

### Materials and growth conditions

*Arabidopsis thaliana* ecotype Columbia-0 was used as the wild type. The T-DNA insertion mutants *bak1-1* (CS6125), *bak1-3* (SALK_034523), *bak1-4* (SALK_116202), *bsk3-1* (SALK_096500), *serk1* (SALK_053021), *serk2* (SALK_058020C), *bak7* (SALK_058093), and *bak8* (SALK_147275C) were acquired from the SALK Institute. The other mutants, *bri1-301* [43], *bzr1-D* [44], *bes1-D* [45], *sud1* [46], and the transgenic plants overexpressing *BRI1* [47], *DWF4* [48], and pBAK1:BAK1-6xHA [49] were also acquired from the SALK Institute. The pBAK1:BAK1/*bak1-4* plants were obtained from Dr. Zipfel’s group [50] and *fer4* mutant seeds were gently provided by Dr. A. Cheung from University of Massachusetts, Amherst. Plants overexpressing AtRALF1 were generated previously [3]. *A*. *thaliana* seeds were surface sterilized in a 50% (v/v) bleach solution for 20 min and washed 5 times using sterile Milli-Q water. Sterilized seeds were stratified for 72 h at 4 °C and then germinated on soil or in plates containing medium. The medium used in all experiments was half-strength MS medium [51] (MS salts without sucrose and vitamins, PhytoTechnology Laboratories), adjusted to pH 5.8 using KOH with or without 6 g l^-1^ Phytagel (Sigma). Plates were grown in a Conviron controlled environmental growth chamber (16 h light, 150 μmol m^–2^ s^–1^ and 8 h dark) at 22 ± 2 °C or in a growth room under the same light conditions. Wild-type *Nicotiana benthamiana* plants for agroinfiltration experiments were grown on soil under 14 h light and 10 h dark at 23–25 °C.

### Production of recombinant proteins

The AtRALF1 and AtRALF34 coding regions were amplified from genomic DNA by conventional PCR using specific primers (S1 Table). The amplified fragments were cloned into the pET-28b expression vector at the *Nde*I */Hind*III restriction sites. The coding region of the extracellular domain (ECD) of the LRR-RLK protein BAK1 was amplified and cloned into the *Nde*I/*Bam*HI sites of the pGADT7 vector (Clontech Laboratories) fused to a hemagglutinin (HA) epitope tag at the N-terminus. This coding region of the extracellular domain of the BAK1 protein fused to the HA epitope tag was amplified by conventional PCR using the primers HA-BAK1-ECD Fw and HA-BAK1-ECD Rv. The amplified fragment, named HA-BAK1-ECD, was cloned into the pENTR D-TOPO vector and later transferred by recombination to the pDEST17 vector. The proteins were fused to a histidine tag at their N-termini. Vector sequences were confirmed by DNA sequencing. The vectors were introduced into the *Escherichia coli* strain Rosetta DE3 or BL21. The recombinant proteins were produced, purified and analyzed as described previously [5]. HPLC-purified AtRALF1 (8 mg) was used as standard to perform HPLC runs with increasing concentrations (25, 50, 100, 200 and 400 μg) of the peptide (S21A Fig). The peak area corresponding to each AtRALF1 concentration was used to stablish a regression line that was used for further quantifications of recombinant AtRALFs (S21B Fig).

### Root growth inhibition assay

Aliquots of 1 ml of medium containing seeds were distributed into 24-well plates. The peptides (10 μM) were added after 2 days, and the root length was measured 2 days after treatment. To maximize root growth, the seeds were germinated in vertical plates. Plates were incubated in a growth room under the same conditions for 5 days, and the seedlings were then transferred to vertical plates containing the same medium with different concentrations of AtRALF1, or 0.1 μM 6-benzylaminopurine (BAP), or 0.1 μM 1-naphthaleneacetic acid (NAA) or 1.0 μM brassinolide (BL). The root length was measured 8 days after treatment unless stated otherwise. Control plants were grown under identical conditions in a peptide-free medium. Quantitative data were obtained as previously described. The experiments were repeated at least 3 times using independent biological replicates with similar results. The data from one representative experiment are shown. For cell length measurements, AtRALF1 peptide (10 μM) was added after 2 days of germination, and the cell length was measured after 2 days of treatment. The cell wall was stained with a 1 mg ml^-1^ propidium iodide solution (Sigma) in the dark for 15 min and washed twice with water. Cells of the endodermis of the root differentiation zone were imaged and measured using confocal microscopy (Olympus FV1000). Approximately 30 plants from each genetic background and at least 5 cells per root were measured. The wavelengths for excitation and emission were 555 and 655 nm, respectively. Image processing was completed using the Olympus FluoView software.

### Gel containing the pH-sensitive indicator bromocresol purple

To analyze the rhizosphere alkalinization, seedlings were grown in plates for 7 days. To avoid damage to the roots, the medium was solidified with 12 g l^-1^ Phytagel. Seedlings were transferred to plates containing the pH indicator bromocresol purple (pH 5.7) as described previously [4,52] and treated with 10 μM AtRALF1 or water. Images were captured 30 min after transfer onto the gel indicator. The experiments were repeated at least three times using independent biological replicates. The data shown are from one representative experiment.

### Lithium sensitivity assay

This experiment was performed as previously described [10]. Seedlings were grown in vertical plates for 3 days, transferred to the same medium in the presence of 18 mM LiCl, and incubated for 4 days. Control plants were grown under identical conditions in LiCl-free medium. The experiments were repeated three times using independent biological replicates. The data shown are from one representative experiment.

### Ca^2+^ mobilization assay

The cytoplasmic Ca^2+^ assay was performed in *Arabidopsis* seedlings as described previously [11]. Changes in Ca^2+^ levels were measured using an *Arabidopsis* line homozygous for the single insertion of a transgene encoding a cytoplasmically expressed apoaequorin protein driven by the constitutive promoter 35S [53], here denoted Wt-aeq. Wt-aeq plants were crossed with *bak1-3* mutants, and T3 generation plants overexpressing apoaequorin and containing a T-DNA insertion in the *BAK1* gene, named *bak1-3*-aeq, were selected and evaluated using semi-quantitative RT-PCR with specific primers (S1 Table). *Arabidopsis* seedlings were grown in plates for 4 days. A single seedling was transferred into each well of a 96-well white microplate (Thermo Labsystems) containing 200 μl of medium supplemented with 2.5 mM coelenterazine cp (Biotium) and incubated in the dark at 24 °C for 16 h. Each well containing a single seedling received recombinant AtRALF1 or acridinium-labeled AtRALF1 at a final concentration of 5, 10 or 100 nM as indicated. The resulting luminescence emission was monitored using a microplate reader (Biotec ELx 800) and 20 time-points for approximately 160 s. The experiments were repeated at least three times using independent biological replicates. The data shown are from one representative experiment.

### Production and analysis of hybrid *Arabidopsis* plants

*A*. *thaliana* plants overexpressing *AtRALF1* (p35S:AtRALF1) were crossed with *bak1-4* mutants as previously described [54]. Semi-quantitative RT-PCR was used to select T3-generation plants overexpressing *AtRALF1* and containing a T-DNA insertion in the *BAK1* gene. The expression levels of *AtRALF1* were monitored by protein blotting using anti-AtRALF1 antibody. The AtRALF1 peptide was purified from seven-day-old seedlings (~2000) as previously described [3]. Homozygous plants accumulating AtRALF1 and containing a T-DNA insertion in the *BAK1* gene were used in the experiments and crossed with wild-type plants to obtain F1 descendants for phenotypic analysis.

### AtRALF1 treatment and semi-quantitative RT-PCR analysis

*Arabidopsis* plants were grown for 10 days in vertical plates and then treated in liquid medium containing 1 μM AtRALF1 for 1 h at room temperature. Control plants were incubated under identical conditions in a peptide-free medium. The total RNA was isolated from the roots of *Arabidopsis* plants using TRIzol reagent according to the manufacturer’s instructions (Life Technologies). The cDNA was synthesized using the ImProm-II Reverse Transcription System (Promega) and 1 μg of RNA. An aliquot of cDNA (1.5 μl) was used as the template in the PCR reaction, which was performed for 29 cycles, unless indicated otherwise, using gene-specific primers (S1 Table). The glyceraldehyde 3-phosphate dehydrogenase (*GAPDH*) gene was used as an internal control. All experiments were repeated at least three times with independent biological replicates. The data shown are from one representative experiment.

### qRT-PCR analysis

The qRT-PCR analysis was performed using 10-fold-diluted cDNA, Maxima SYBR Green Rox/qPCR Master Mix (Thermo Scientific) and a StepOne Real-Time PCR System (Applied Biosystems). The glyceraldehyde 3-phosphate dehydrogenase (*GAPDH*) gene was used as an internal control. The primers used are described in S1 Table. The threshold cycle (CT) was determined by the instrument, and the ΔΔCT method was used to calculate the fold change in each gene [55]. An arbitrary value of 1 was attributed to the control treatments. Three replicates were analyzed for each biological sample, and two biological samples were analyzed. The data shown are from one representative experiment.

### Quantification of green fluorescent protein (GFP) in pBAK1:BAK1-GFP transgenic plants

Transgenic *Arabidopsis* plants were grown for 4 days in liquid medium and treated with 1 μM AtRALF1 for 4 h. After 4 h of agitation at room temperature, the intensity of GFP in the roots was analyzed by confocal microscopy (Olympus FV1000). Control plants were kept in a peptide-free medium. The GFP excitation wavelength was 488 nm, and the emission wavelength was 540 nm. Images were generated by a scanner depth of 7 μm. Ten slices per root were generated and compacted in one image. Image processing was performed using the Olympus FluoView software. An average of 20 seedlings were observed under the microscope in at least 3 different experiments using independent biological replicates.

### Production of pAtRALF1-AtRALF1:GFP transgenic plants

The *AtRALF1* gene with its endogenous promoter sequence was amplified from the genomic DNA of *Arabidopsis* using standard PCR with the primers pAtRALF1-RALF1 Fw and pAtRALF1-RALF1 Rv. The fragment was first cloned into the pENTR/D-TOPO vector and transferred by recombination into the pk7FWG2 vector [56]. The pAtRALF1-AtRALF1:GFP sequence (2556 bp) was amplified with the primers pAtRALF1-RALF1 Fw and T35S Rv, cloned into the pENTR/D-TOPO vector, and later transferred by recombination into the pkGWFS7 vector [56]. The construct was verified by DNA sequencing. The *Agrobacterium tumefaciens* strain GV3101 expressing the pkGWFS7 vector containing the pAtRALF1-AtRALF1:GFP insertion was used for the stable transformation of *Arabidopsis* using the floral dip method [57]. Transgene expression was confirmed using semi-quantitative RT-PCR. All primers are listed in S1 Table. Transgenic lines from the T3 generation were used in the experiments.

### Confocal analysis

Transgenic pAtRALF1:AtRALF1-GFP and pBAK1:BAK1-GFP *Arabidopsis* plants were grown for 4 days in liquid medium. The transgenic pBAK1:BAK1-GFP *Arabidopsis* plants were provided by Cyril Zipfel [58]. Seedlings were stained in a 1 mg ml^-1^ propidium iodide solution (Sigma) in the dark for 15 min and washed twice with water. Roots were visualized using confocal microscopy (Olympus FV1000). The wavelengths for GFP excitation and emission were 488 nm and 540 nm, respectively, and the wavelengths for propidium iodide excitation and emission were 555 and 655 nm, respectively. Image processing was completed using the Olympus FluoView software.

### Two-hybrid system in yeast

The coding regions of the mature peptides AtRALF1 and AtRALF34 were amplified from *Arabidopsis* genomic DNA and fused to the GAL4 DNA-binding domain and the MYC epitope tag using the *Nde*I/*Bam*HI sites in the pGBKT7 (Clontech) vector. The coding regions of the extracellular domain of the LRR-RLK proteins BAK1, BAK7, BRI1 and FLS2 were amplified from cDNA and fused to the GAL4 DNA-activation domain and HA epitope tag using the *Nde*I/*Bam*HI sites in the pGADT7 (Clontech) vector. Deletions of the extracellular domain of BAK1 were obtained using PCR and specific primers. The amplified fragments were fused to the GAL4 DNA-activation domain and the HA epitope tag using the *Nde*I/*Bam*HI sites in the pGADT7 (Clontech) vector. All resulting plasmids were verified by sequencing. The primers used to amplify the fragments are listed in S1 Table. The desired pairs of the pGBKT7 and pGADT7 vectors (Clontech) were co-transformed into yeast strain AH109 as described [59]. Transformed yeast cells were selected on a synthetic complete medium lacking leucine, tryptophan and histidine. To detect the proteins, transformed yeast cells were inoculated into synthetic complete medium [60] and grown overnight at 28 °C, and the proteins were extracted as previously described [60]. The proteins fused to the HA and MYC epitope tags were detected by protein blotting. Anti-HA antibody (1:1000 dilution, Thermo Scientific) and anti-MYC (1:5000 dilution, Life Technologies) were used with the Super Signal West Pico Maximum Chemiluminescent detection kit (Thermo Scientific).

### Co-immunoprecipitation *in vitro* and detection of the proteins

The purified recombinant proteins AtRALF1 and HA-BAK1-ECD and the synthetic peptides RALF1(9–49) and RALF1(I6A) were combined in 200 μl of interaction buffer pH 6.3 (100 mM NaCl; 1 mM PMSF; 0.5%Triton X; 2.7 mM KCl; 8 mM NaH_2_PO_4_; 2 mM KH_2_PO_4_) overnight at 4 °C. A 100-nM concentration of each protein was used. This solution was incubated with agarose anti-HA beads (EZview Red Anti-HA Affinity Gel, Sigma) for 30 min at 4 °C to immunoprecipitate the proteins, washed 5 times with interaction buffer, eluted in 1X SDS sample buffer containing 10% β-mercaptoethanol and applied to an SDS-PAGE gel. The immunoprecipitated HA-BAK1-ECD and co-immunoprecipitated AtRALF1 were analyzed by protein blotting as described. The primary and secondary antibodies used to detect AtRALF1 were anti-AtRALF1 (1:10,000, Celula B, Immunology Laboratory, UFRGS, Brazil) and goat anti-rabbit HRP-conjugated secondary antibody (1: 10,000, BioRad), respectively.

### Co-immunoprecipitation of AtRALF1 in the microsomal fraction of *Nicotiana benthamiana* leaves overexpressing BAK1-GFP

This assay was performed as previously described with some modifications [10]. The BAK1 coding region was amplified from *Arabidopsis* genomic cDNA using standard PCR with the primers BAK1 Fw and BAK1 Rv (S1 Table). To generate the p35S-BAK1:GFP plasmid, the fragment was cloned into the pENTR D-TOPO vector and later transferred to the pk7FWG2 vector [56]. Leaves from *N*. *benthamiana* plants were agroinfiltrated with p19 vector alone and p19 co-expressed with p35S-BAK1:GFP, as previously described [61]. After 3 days, leaves were homogenized in extraction buffer [300 mM sucrose, 100 mM Tris-HCl pH 7.5, 25 mM EDTA, 25 mM NaF, 1 mM Na_2_MoO_4_, 1 mM PMSF, 1 mM DTT and protease inhibitor cocktail (Roche), pH 6.2 adjusted] and centrifuged at 1,000 g for 10 min at 4°C, and the supernatant was then centrifuged at 100,000 g for 50 min at 4°C to precipitate the microsomal pellet. The pellet was resuspended in the extraction buffer plus 0.5% Triton-X and incubated with 600 nM recombinant AtRALF1 or 600 nM RALF1(I6A) for 1 h at 4°C. The complexes were incubated with anti-GFP antibody (Santa Cruz Biotechnology) and Sepharose Beads plus protein A (Life Technologies) overnight at 4°C. The unbound materials were removed by washing 5 times. The AtRALF1/BAK1-GFP complex bound to the beads was eluted with denaturing in 10 μl of 1X loading buffer (NuPAGE LDS buffer) at 95°C for 8 min, then analyzed by SDS-PAGE. The proteins were detected as previously described.

### Isothermal titration calorimetry (ITC)

ITC was performed using a VP ITC with a 1.4 ml standard cell and a 293 μl titration syringe. Proteins were dissolved in ITC buffer (15 mM HEPES (4-(2-hydroxyethyl)-1-piperazineethanesulfonic acid, pH 6.8) and the experiments were performed at 25°C. Molar protein concentration for BAK1-ECD and AtRALF1 were calculated using their molecular weight of 25,654.04 and 7,631 Da, respectively (determined by HPLC). The concentrations for the complex titrations were 0.7 mM of protein (BAK1-ECD) in the syringe and 0.03 mM of ligand (AtRALF1) in the cell at time intervals of 300 s (28 injections of volume 10 μl) to ensure that the titration peak returned to the baseline.

ITC data were corrected for the heat of dilution by subtracting the mixing enthalpies for titrant solution injections into protein free ITC buffer. Data were analyzed using the Origin software (version 7.0) as provided by the manufacturer. Independent experiments were repeated three times with similar results.

### Production of acridinium-labeled AtRALF peptides

Acridinium esters were conjugated to the N-terminal of the peptides AtRALF1 and AtRALF34 according to the manufacturer’s instructions (Cayman Chemical). The acridinium-labeled peptides (acriAtRALF1 and acriAtRALF34) were separated from the unlabeled peptides by HPLC using a semipreparative reversed-phase C18-HPLC column (Kromasil). A gradient from 0 to 40% acetonitrile/0.1% formic acid and a flow rate of 1 ml min^-1^ were used. The isolated acridinium-labeled peptides were lyophilized, and the resulting powder was dissolved in water. To quantify the purified acriAtRALF1, a C18 reversed-phase narrow-bore HPLC column (Kromasil) was used. A gradient from 0 to 100% acetonitrile over 60 min and a flow rate of 0.2 ml min^-1^ was used.

### Binding assay using acridinium-labeled AtRALF peptides

*Arabidopsis* seedlings were grown for 5 days on vertical plates. Roots were homogenized in extraction buffer [300 mM sucrose, 100 mM Tris-HCl pH 7.5, 25 mM EDTA, 25 mM NaF, 1 mM Na_2_MoO_4_, 1 mM PMSF and protease inhibitor cocktail (Roche), adjusted to pH 6.2], and the homogenate was spun at 2,000 x g for 10 min at 4 °C. The supernatant was recovered and centrifuged at 100,000 x g for 50 min at 4 °C to precipitate the microsomal pellet. The pellet was resuspended in the extraction buffer plus 0.5% Triton-X. Seedlings or microsomal fractions were placed in 200 μl of medium and incubated with 7 nM acridinium-labeled peptide (acriAtRALF1 or acriAtRALF34) and different concentrations of AtRALF1, AtRALF34 or RALF1(9–49) for 15–20 min at 4 °C on a shaker. After incubation, the seedlings or microsomal fractions were washed three times with the same medium. Unless indicated otherwise, five entire seedlings and approximately 10 ng of microsomal fractions were used per treatment. The resulting luminescence emissions from the acridinium were measured using a microplate reader (Biotec ELx 800) at 2 s after injecting 50 μl of a solution containing 20 mM H_2_O_2_ in 0.1 M NaOH. These experiments were repeated at least three times using independent biological replicates. Duplicates were used for each concentration or treatment. The data shown are from one representative experiment.

### Phosphorylation assay

Transgenic plants pBAK1:BAK1-6xHA were grown in vertical plates for 10–15 days. The plants were treated with 0.1 μM BL, 2 μM RALF1(9–49), 1 μM, 2 μM or 10 μM AtRALF1 for 20 min. The roots were isolated, and the microsomal fractions were obtained as described above. The microsomal fractions were incubated with anti-HA beads (EZview Red Anti-HA Affinity Gel, Sigma) for 2 h at 4 °C to immunoprecipitate the BAK1 protein. The unbound materials were removed by washing 3 times. Proteins were separated by SDS/PAGE and further analyzed by protein blotting using anti-HA (Thermo Scientific), or anti-phosphothreonine (Novus Biologicals), or anti-phosphoserine (Novus Biologicals) antibodies.

## Supporting information

S1 Fig Loss-of-function *bak1-1* and *bak1-3* mutants are insensitive to the inhibitory activity of AtRALF1 on root growth. *Arabidopsis* seedlings (2-days-old) were treated with 10 μM AtRALF1 or water (H_2_0) for 2 days. The values are the mean ± SD of at least 25 seedlings. Triple asterisks indicate P < 0.01 (Student’s t test); ns, not significant.
(PDF)

S2 Fig Loss-of-function *bak1* mutants are sensitive to the inhibitory activity of AtRALF23/34 on root growth. *Arabidopsis* seedlings (2-day-old) were treated with 10 μM RALF1(9–49), RALF1(I6A), AtRALF1, AtRALF23 and AtRALF34 or water (H_2_0) for 2 days. The values are the mean ± SD of at least 20 seedlings. The means with the same letter are not significantly different from each other (Tukey’s test, P ≤ 0.01).
(PDF)

S3 Fig *BAK1* T-DNA Insertion Lines Validation. (A) T-DNA insertion sites of the knockout lines *bak1-1 (CS6125)*, *bak1-3 (SALK_034523)* and *bak1-4 (SALK_116202)*. UTR, untranslated region. (B) Validation of the T-DNA insertion in the *Arabidopsis* genome of the lines *bak1-1*, *bak1-3* and *bak1-4*. T-DNA, amplification products of a PCR reaction using the T-DNA specific primer LBb1.3 and one gene-specific primer. GSP, amplification products of a PCR reaction using two gene-specific primers. The detection of the AtRALF34 gene was used as an internal control. (C) RT-PCR analyses to confirm the mutants. *GAPDH* gene expression was used as internal control. Wt, wild-type.
(PDF)

S4 Fig Analyses of root growth of *Arabidopsis* seedlings treated with AtRALF1 peptide for 8 days and evaluated daily. Four-day-old seedlings were transferred to media containing different concentrations of AtRALF1, and the primary root length was measured daily during the 8 days of treatment. Genotypes Wt, *bak1-1*, *bak1-3* and *bak1-4* are indicated on top of each series of graphics. The seedlings were treated with 0, 0.5, 1, 5 and 10 μM AtRALF1. The data represent the mean values ± SD of 30 seedlings. Double and triple asterisks indicate P < 0.05 and P <0.01, respectively (Student’s t test); ns, not significant.
(PDF)

S5 Fig Loss-of-function *bak7* mutants are sensitive to the inhibitory activity of AtRALF1 on root growth. Four-day-old seedlings were transferred to medium containing 10 μM AtRALF1, and the primary root length was measured after 8 days of treatment. The data represent the mean value ± SD of 20 seedlings. Triple asterisks indicate P < 0.01 (Student’s t test); ns, not significant. Representative *Arabidopsis* seedlings after 8 days of treatment with AtRALF1 are shown. The seedlings were arranged on plates after the treatment for imaging. Scale bars, 1 cm.
(PDF)

S6 Fig Loss-of-function *bak1* mutants are sensitive to the inhibitory activity of the cytokinin BAP on root growth. Four-day-old seedlings were transferred to medium containing 0.1 μM BAP, and the primary root length was measured after 8 days of treatment. The data represent the mean value ± SD of 10 seedlings. Triple asterisks indicate P < 0.01 (Student’s t test); ns, not significant. Representative *Arabidopsis* seedlings after 8 days of treatment with BAP are shown. The seedlings were arranged on plates after the treatment for imaging. Scale bars, 1 cm.
(PDF)

S7 Fig Loss-of-function *bak1* mutants are sensitive to the inhibitory activity of auxin NAA on root growth. Four-day-old seedlings were transferred to medium containing 0.1 μM NAA, and the primary root length was measured after 8 days of treatment. The data represent the mean value ± SD of 10 seedlings. Triple asterisks indicate P < 0.01 (Student’s t test); ns, not significant. Representative *Arabidopsis* seedlings after 8 days of treatment with NAA are shown. The seedlings were arranged on plates after the treatment for imaging. Scale bars, 1 cm.
(PDF)

S8 Fig Loss-of-function *bak1* mutants have reduced sensitivity to brassinolide (BL). Four-day-old seedlings were transferred to medium containing 0 or 1μM BL, and the primary root length was measured after 8 days of treatment. The data represent the mean value of two experiments with +- SD of 15 and 10 seedlings in each one. Representative Arabidopsis seedlings after 8 days of treatment with BL are shown. The seedlings were arranged on plates after the treatment for imaging. Scale bars, 1cm.
(PDF)

S9 Fig *bak1* mutants are insensitive to high concentration of AtRALF1. As opposed to *fer4* mutant, that is insensitive to AtRALF1 peptide in low concentrations only, *bak1* mutants are insensitive to AtRALF1 concentrations as high as 10 μM. Four-day-old seedlings were transferred to media containing different concentrations of AtRALF1, and the primary root length was measured after 8 days of treatment. The data represent the mean value ± SD of 30 seedlings.
(PDF)

S10 Fig AtRALF1 is localized at the root endodermis. Confocal microscopic analysis of AtRALF1 peptide fused to GFP in roots of pAtRALF1:AtRALF1-GFP transgenic plants. (A) AtRALF1-GFP expression in the meristematic zone (MZ), elongation zone (EZ) and differentiation zone (DZ). Bars, 100 μm. (B) AtRALF1-GFP expression in the endodermis layer (EL). Bars, 25 μm. PI, propidium iodide.
(PDF)

S11 Fig BAK1 is localized at the endodermis, cortex and epidermis of the root. Confocal microscopic analysis of BAK1 peptide fused to GFP in roots of pBAK1:AtBAK1-GFP transgenic plants. (A) BAK1-GFP expression in the meristematic zone (MZ), elongation zone (EZ) and differentiation zone (DZ). Bars, 100 μm. (B) BAK1-GFP expression in endodermis layer (EL), cortex (C) and epidermis layer (EP). Bars, 25 μm. PI, propidium iodide.
(PDF)

S12 Fig Analyses of protein extracts from yeast. (A) Blot of HA-tagged proteins: BAK1-ECD:HA, BAK7-ECD:HA, BRI1-ECD:HA, and FLS2-ECD:HA. ECD, extracellular domain. The proteins were monitored using an anti-HA antibody. (B) Blot of the MYC-tagged protein AtRALF1 using an anti-MYC antibody. (C) Blot of HA-tagged truncated proteins: **1**, LRR1 + LRR2 + LRR3 + LRR4 + LRR5 + Prorich; **2**, LRR2 + LRR3 + LRR4 + LRR5 + Prorich; **3**, LRR3 + LRR4 + LRR5 + Prorich; **4**, LRR4 + LRR5 + Prorich; **5**, LRR5 + Prorich; **6**, Pro-rich; **7**, Leu zippers + LRR1 + LRR2 + LRR3 + LRR4 + LRR5; **8**, Leu zippers + LRR1 + LRR2 + LRR3 + LRR4; **9**, Leu zippers + LRR1 + LRR2 + LRR3; **10**, Leu zippers + LRR1 + LRR2; **11**, Leu zippers + LRR1; **12**, Leu zippers. The truncated proteins were monitored using an anti-HA antibody. EP, empty vector. Empty vectors were used as negative controls.
(PDF)

S13 Fig The AtRALF34 peptide does not interact with the extracellular domain of BAK1, BAK7, BRI1 and FLS2 in yeast. (A) The active AtRALF34 peptide fused to the GAL4 DNA-binding domain (AtRALF34-pGBKT7) was used as bait to test its interaction with the extracellular domain (ECD) of the proteins fused to the GAL4 activation domain (pGADT7): BAK1 (BAK1-ECD), BAK7 (BAK7-ECD), BRI1 (BRI1-ECD) and FLS2 (FLS2-ECD). Transformed yeast cells were selected on a synthetic complete medium lacking leucine, tryptophan and histidine to test interactions. (B) Analyses of protein extract from yeast: blot of the protein AtRALF34 using an anti-RALF antibody. The empty vector was used as negative control.
(PDF)

S14 Fig Recombinant HA-BAK1-ECD protein analysis. (A) The chromatographic profile of HA-BAK1-ECD recombinant protein eluted from a C-18 narrow-bore HPLC column. Dashed line shows the percentage of acetonitrile during the analysis. Approximately 50 μg of the protein was used. (B) Mass spectrometric analysis of trypsin-digested HA-BAK1-ECD protein. The mass of three tryptic fragments (1313.7; 2305.3 e 2444.5) obtained confirmed the identity of the peptide. The theoretical sequences are presented and underlined sequences indicate the identified fragments. No alterations in the HA-BAK1-ECD protein were detected.
(PDF)

S15 Fig Isothermal titration calorimetry (ITC) of AtRALF1 and BAK1-ECD. (A) AtRALF1 in 15mM HEPES, pH 6.8. ITC experiments were simulated using the following parameters: 28 injections of AtRALF1 (0.7 mM, volume 10 μL) in HEPES (volume cell = 1.4 mL) at 25°C. (B) BAK1-ECD in 15mM HEPES, pH 6.8. ITC experiments were simulated using the following parameters: 28 injections of BAK1-ECD (0.7 mM, volume 10 μL) in HEPES (volume cell = 1.4 mL) at 25°C. (C) HEPES in AtRALF1. ITC experiments were simulated using the following parameters: 28 injections of buffer 15mM HEPES, pH 6.8 (volume 10 μL) in AtRALF1 (0.03 mM, volume cell = 1.4 mL) at 25°C.
(PDF)

S16 Fig Isothermal titration calorimetry (ITC) of RALF1(9–49) andBAK1-ECD. (A) BAK1-ECD in 0.03 mM RALF1(9–49). ITC experiments were simulated using the following parameters: 28 injections of BAK1-ECD (0.7 mM, volume 10 μL) in RALF1(9–49) (0.03 mM, volume cell = 1.4 mL) at 25°C. (B) HEPES in RALF1(9–49). ITC experiments were simulated using the following parameters: 28 injections of buffer 15mM HEPES, pH 6.8 (volume 10 μL) in RALF1(9–49) (volume cell = 1.4 mL) at 25°C. (C) BAK1-ECD in 15mM HEPES, pH 6.8. ITC experiments were simulated using the following parameters: 28 injections of BAK1-ECD (0.7 mM, volume 10 μL) in HEPES (volume cell = 1.4 mL) at 25°C.
(PDF)

S17 Fig Analyses of acridinium-labeled AtRALF1 (acriAtRALF1) and binding of acriAtRALF1 in *bak1-4 and fer4* mutants. (A) The Ca^2+^ mobilization assay comparing the acriAtRALF1 activity with the AtRALF1 peptide. A total of 20 time-points per treatment were measured over 160 sec and summed. The results are the means ± SD of two measurements. (B) acriAtRALF1 binding in whole seedlings and only in roots of *Arabidopsis*. The roots were excised just before measurements. Ctr, seedlings treated with unlabeled AtRALF1. (C) Whole seedlings (5 days old) were treated for 20 min with acriAtRALF1 (acriR1), acriR1 and an excess of unlabeled AtRALF1 (R1), as indicated. The values are the mean ± SD of three measurements (5 seedlings each). The proportions of labeled: unlabeled proteins are shown in parentheses. Triple asterisks indicates P < 0.001 (Student’s t test).
(PDF)

S18 Fig Binding of acridinium-labeled AtRALF1 and AtRALF34 to intact *Arabidopsis* seedlings. (A) *bak7* seedlings (5 days old) were treated for 15 min with acriAtRALF1 (acriR1), acriR1 and an excess of unlabeled AtRALF1 (R1), as indicated. (B) *bak1* seedlings (5 days old) were treated for 15 min with acriAtRALF34 (acriR34), acriR34 and an excess of unlabeled AtRALF34 (R34) or AtRALF1 (R1), as indicated. The proportions of labeled: unlabeled proteins are shown in parentheses. The values are the mean ± SD of two measurements (5 seedlings each).
(PDF)

S19 Fig BAK1 phosphorylation increases in roots of Arabidopsis after AtRALF1 treatment and shows a dose-dependent response up to 2 μM. pBAK1:BAK1-6xHA plants were treated with water (H_2_0), 2 μM RALF1(9–49) [R9-49], 0.1 μM BL, 1, 2 and 10 μM AtRALF1 for 20 min. After treatment, the microsomal fraction was isolated from roots. BAK1 protein was immunoprecipitated with anti-HA beads and subjected to immunoblot analysis with anti-phospho-Thr and anti-HA antibodies. The numbers indicate the relative intensity of the bands.
(PDF)

S20 Fig BAK1 phosphorylation in roots of *Arabidopsis* after AtRALF1 treatment using anti-phospho-Ser. pBAK1:BAK1-6xHA plants were treated with water (H_2_0), 2 μM RALF1(9–49) [R9-49], 0.1 μM BL, 1, 2 and 10 μM AtRALF1 for 20 min. After treatment, the microsomal fraction was isolated from roots. BAK1 protein was immunoprecipitated with anti-HA beads and subjected to immunoblot analysis with anti-phospho-Ser and anti-HA antibodies.
(PDF)

S21 Fig HPLC quantification of the recombinant AtRALF1. (A) HPLC profiles of the recombinant AtRALF1. The recombinant peptide was extracted from E. coli, affinity purified with a Ni2+ resin, dialyzed against 0.1% formic acid, HPLC purified, lyophilized, resuspended (0.1% formic acid) and injected again into a C18 reversed-phase HPLC column (8-mm 4,6 x 25 cm Kromasil) previously equilibrated with formic acid 0.1%. Increasing concentrations (25, 50, 100, 200 e 400 mg) were loaded into the column and eluted using an acetonitrile gradient (0 to 50% in 30 min). (B) Linear regression between the peak area and the corresponding AtRALF1 concentration. The linear equation and the R2 coefficient is shown.
(PDF)

S1 Table Primers used. (PDF)
