# Supplementary figures and images for: BAK1 is involved in AtRALF1-induced inhibition of root cell expansion
Source: PLoS Genet. 2017 Oct 13;13(10):e1007053. doi: 10.1371/journal.pgen.1007053 (PMC5656322; doi:10.1371/journal.pgen.1007053)

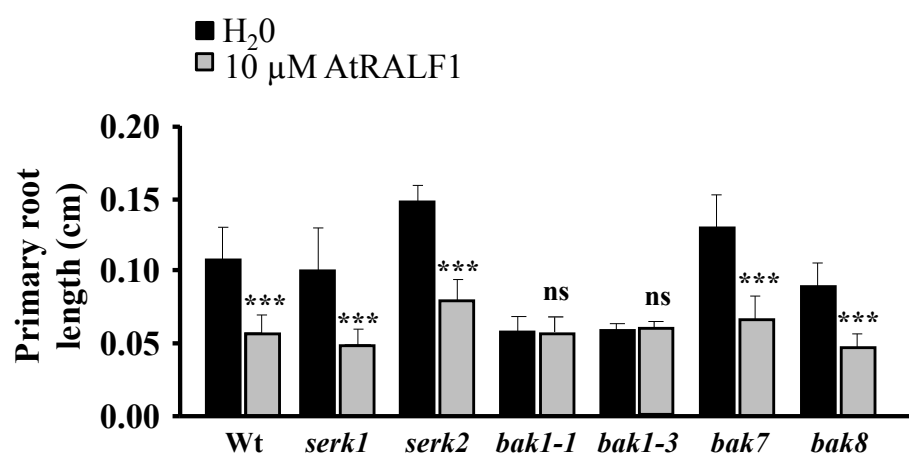

Supplement: S1 Fig — Arabidopsis seedlings (2-days-old) were treated with 10 μM AtRALF1 or water (H20) for 2 days. The values are the mean ± SD of at least 25 seedlings. Triple asterisks indicate P < 0.01 (Student’s t test); ns, not significant. (PDF) [file pgen.1007053.s001.pdf]

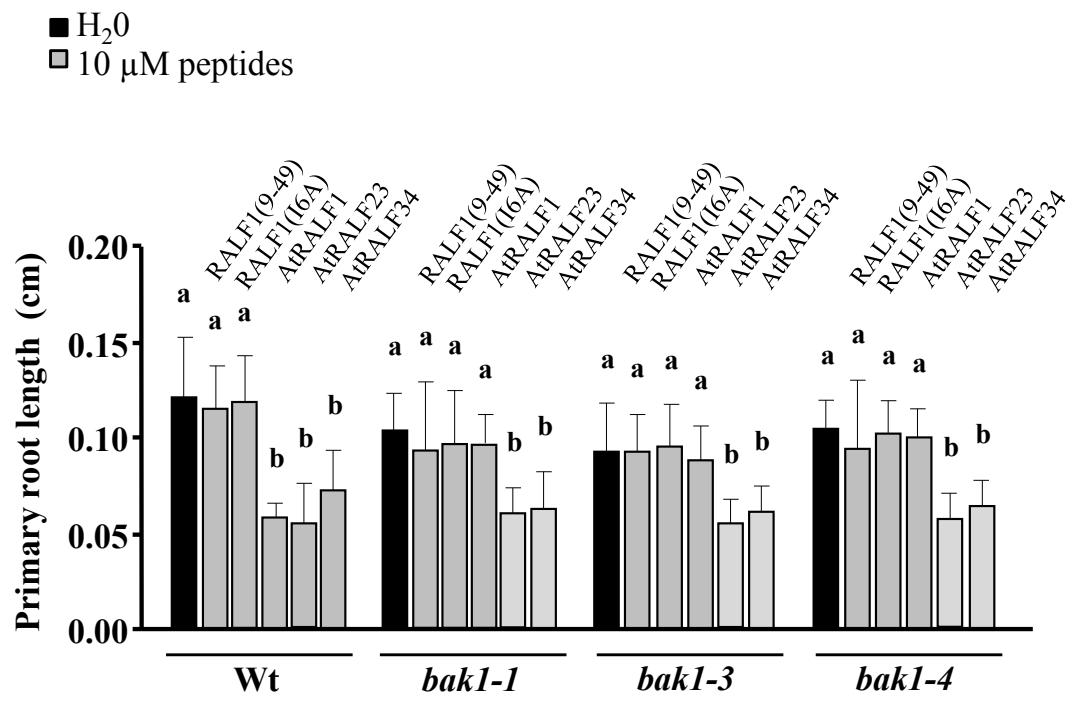

Supplement: S2 Fig — Arabidopsis seedlings (2-day-old) were treated with 10 μM RALF1(9–49), RALF1(I6A), AtRALF1, AtRALF23 and AtRALF34 or water (H20) for 2 days. The values are the mean ± SD of at least 20 seedlings. The means with the same letter are not significantly different from each other (Tukey’s test, P ≤ 0.01). (PDF) [file pgen.1007053.s002.pdf]

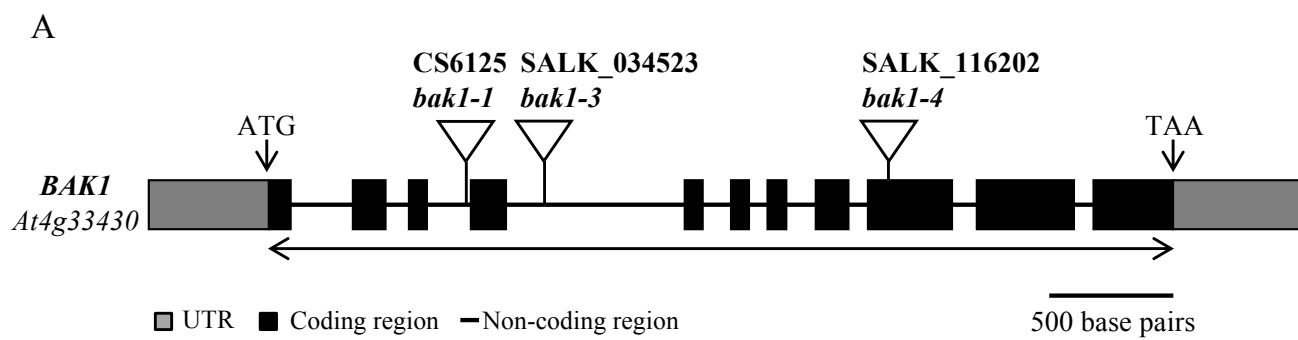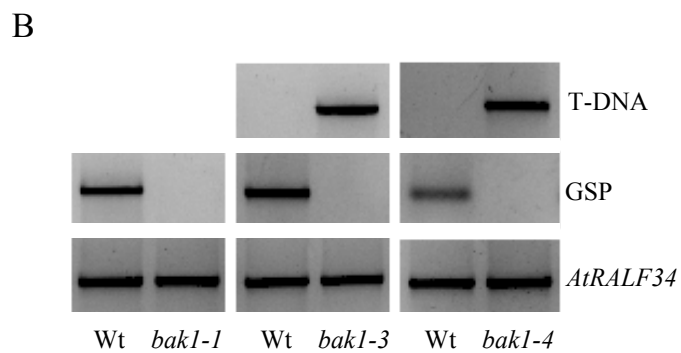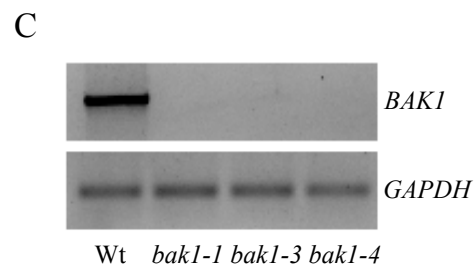

Supplement: S3 Fig — (A) T-DNA insertion sites of the knockout lines bak1-1 (CS6125), bak1-3 (SALK_034523) and bak1-4 (SALK_116202). UTR, untranslated region. (B) Validation of the T-DNA insertion in the Arabidopsis genome of the lines bak1-1, bak1-3 and bak1-4. T-DNA, amplification products of a PCR reaction using the T-DNA specific primer LBb1.3 and one gene-specific primer. GSP, amplification products of a PCR reaction using two gene-specific primers. The detection of the AtRALF34 gene was used as an internal control. (C) RT-PCR analyses to confirm the mutants. GAPDH gene expression was used as internal control. Wt, wild-type. (PDF) [file pgen.1007053.s003.pdf]

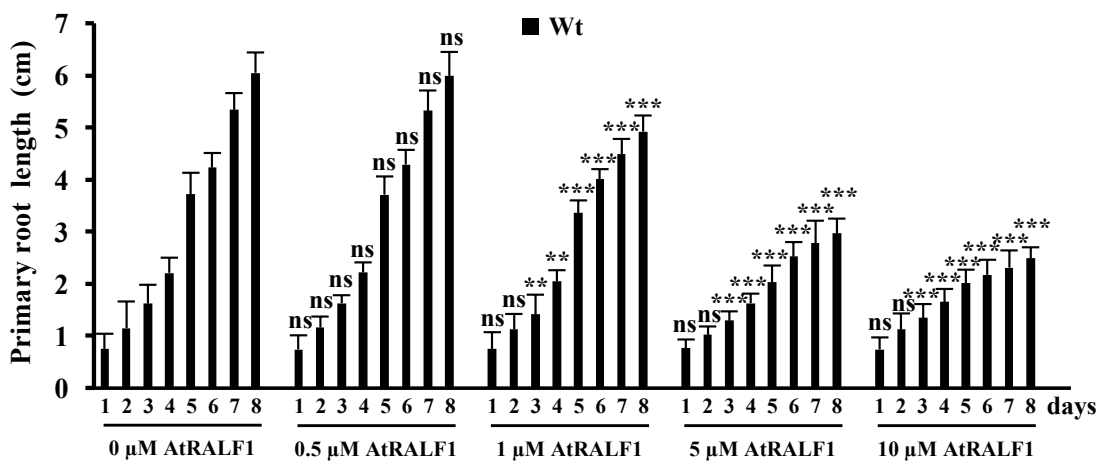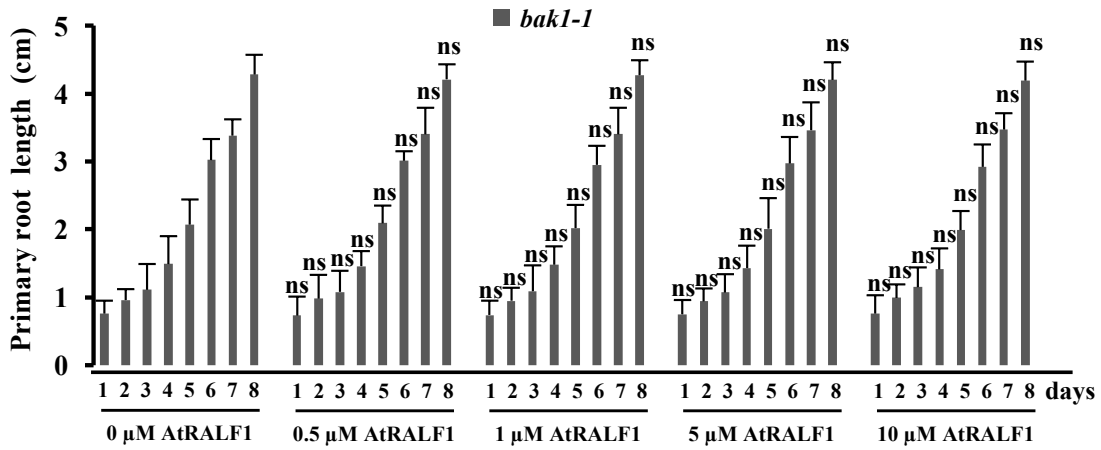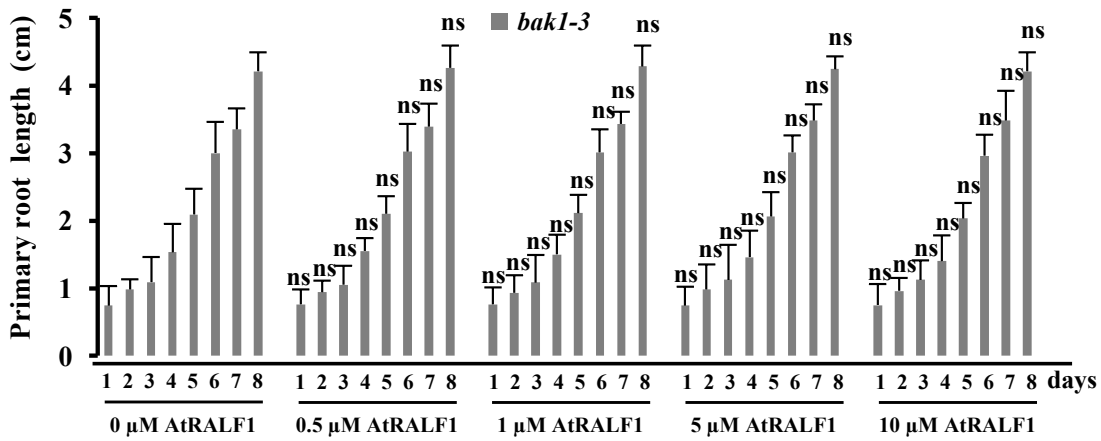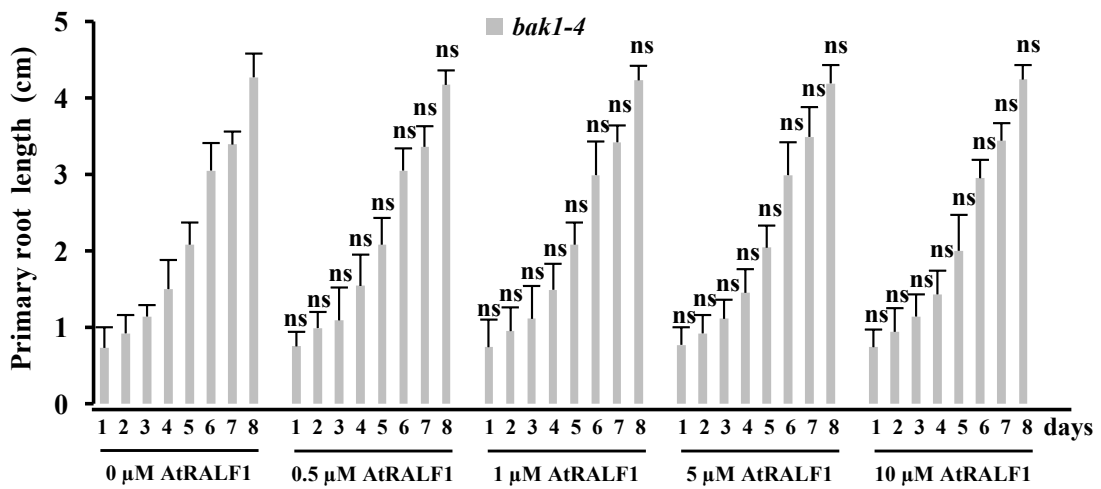

Supplement: S4 Fig — Four-day-old seedlings were transferred to media containing different concentrations of AtRALF1, and the primary root length was measured daily during the 8 days of treatment. Genotypes Wt, bak1-1, bak1-3 and bak1-4 are indicated on top of each series of graphics. The seedlings were treated with 0, 0.5, 1, 5 and 10 μM AtRALF1. The data represent the mean values ± SD of 30 seedlings. Double and triple asterisks indicate P < 0.05 and P <0.01, respectively (Student’s t test); ns, not significant. (PDF) [file pgen.1007053.s004.pdf]

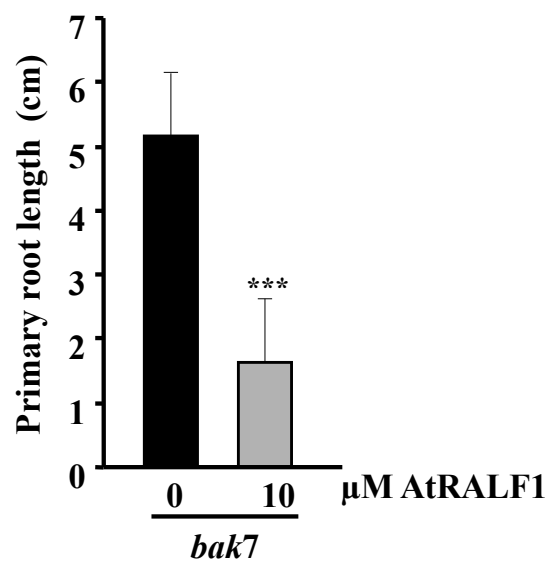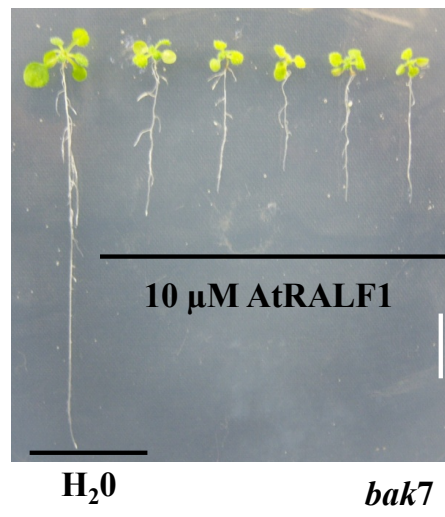

Supplement: S5 Fig — Four-day-old seedlings were transferred to medium containing 10 μM AtRALF1, and the primary root length was measured after 8 days of treatment. The data represent the mean value ± SD of 20 seedlings. Triple asterisks indicate P < 0.01 (Student’s t test); ns, not significant. Representative Arabidopsis seedlings after 8 days of treatment with AtRALF1 are shown. The seedlings were arranged on plates after the treatment for imaging. Scale bars, 1 cm. (PDF) [file pgen.1007053.s005.pdf]

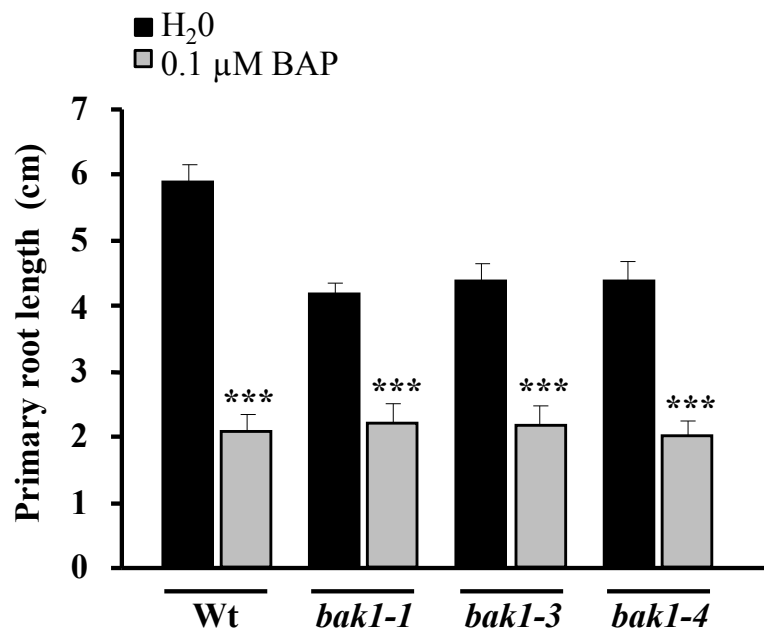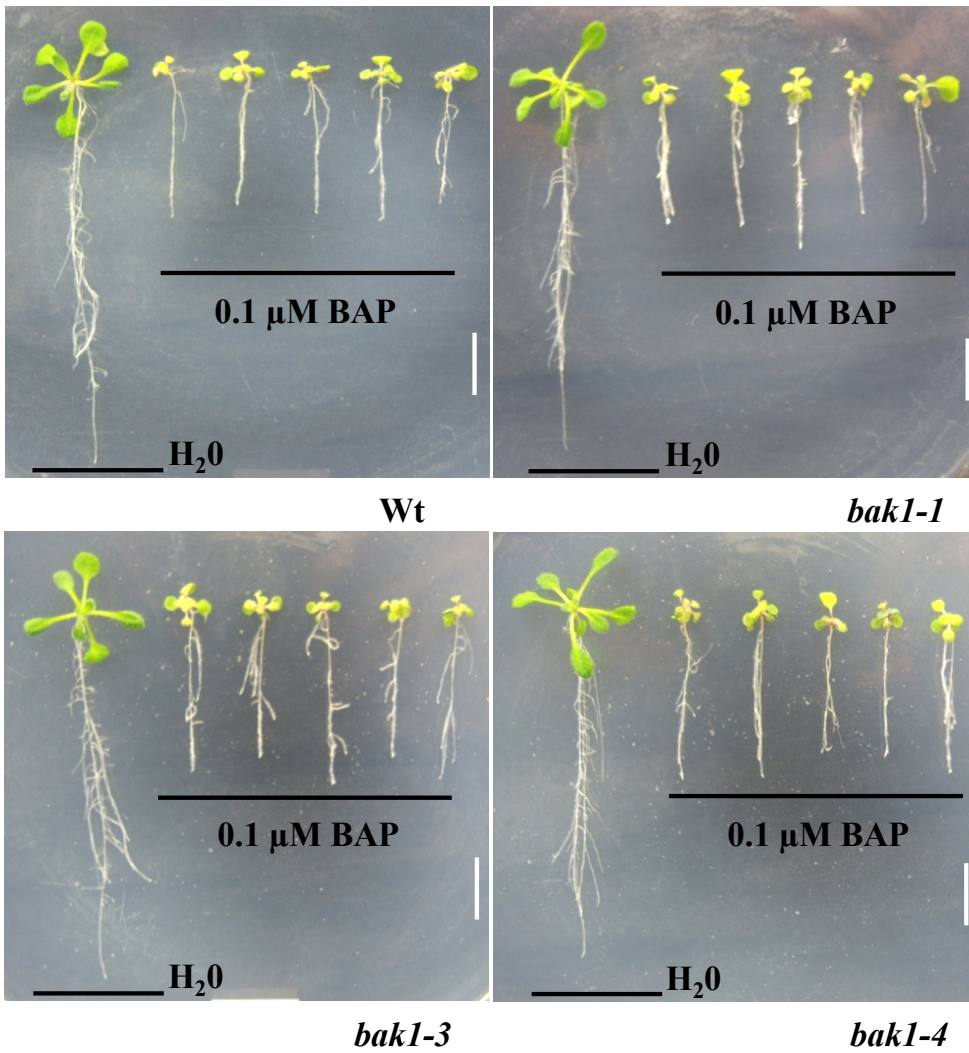

Supplement: S6 Fig — Four-day-old seedlings were transferred to medium containing 0.1 μM BAP, and the primary root length was measured after 8 days of treatment. The data represent the mean value ± SD of 10 seedlings. Triple asterisks indicate P < 0.01 (Student’s t test); ns, not significant. Representative Arabidopsis seedlings after 8 days of treatment with BAP are shown. The seedlings were arranged on plates after the treatment for imaging. Scale bars, 1 cm. (PDF) [file pgen.1007053.s006.pdf]

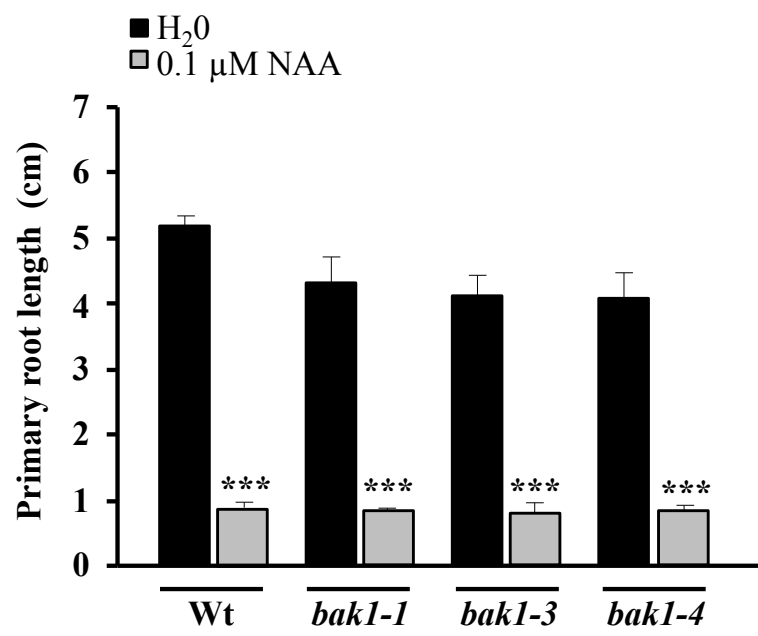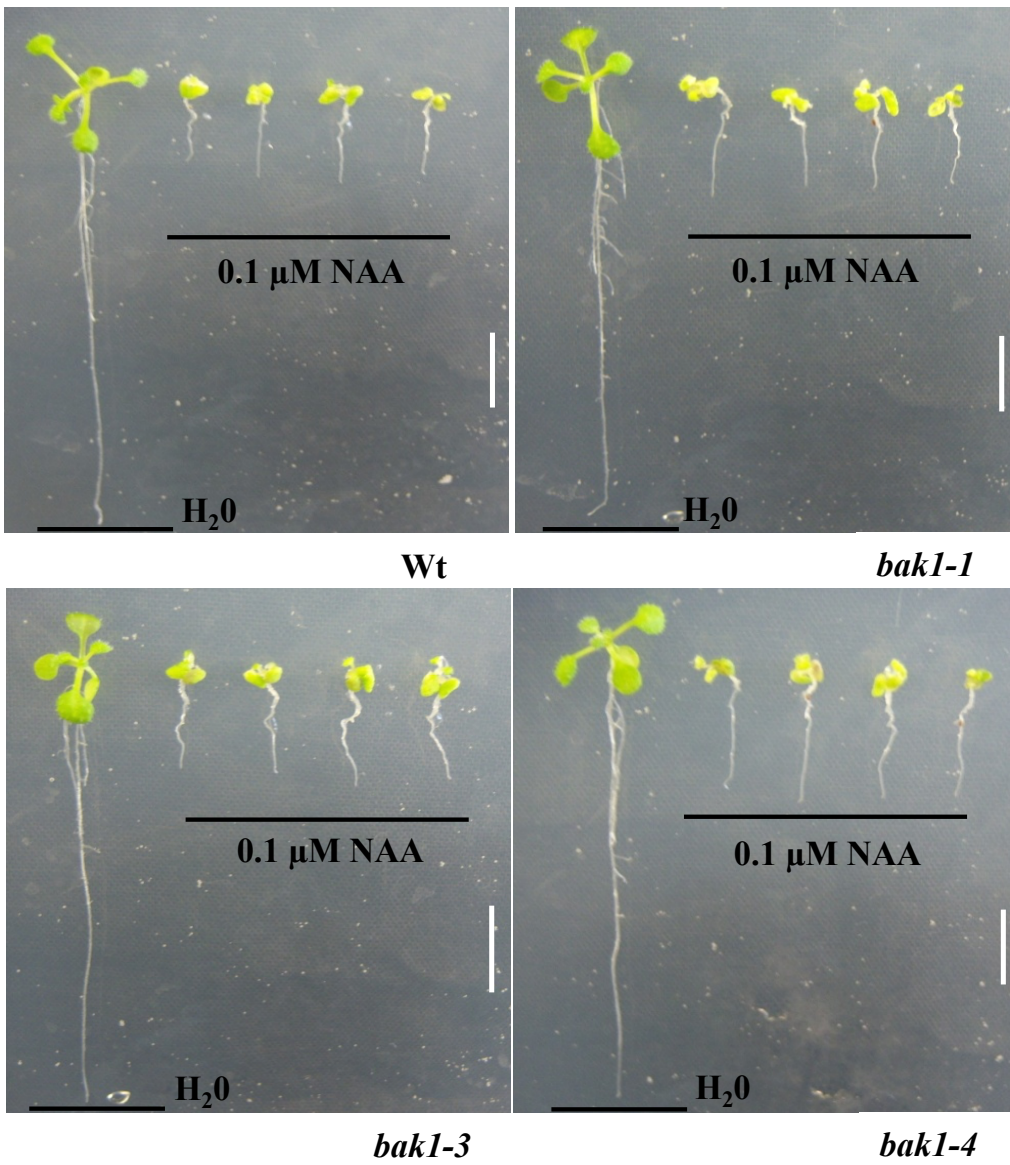

Supplement: S7 Fig — Four-day-old seedlings were transferred to medium containing 0.1 μM NAA, and the primary root length was measured after 8 days of treatment. The data represent the mean value ± SD of 10 seedlings. Triple asterisks indicate P < 0.01 (Student’s t test); ns, not significant. Representative Arabidopsis seedlings after 8 days of treatment with NAA are shown. The seedlings were arranged on plates after the treatment for imaging. Scale bars, 1 cm. (PDF) [file pgen.1007053.s007.pdf]

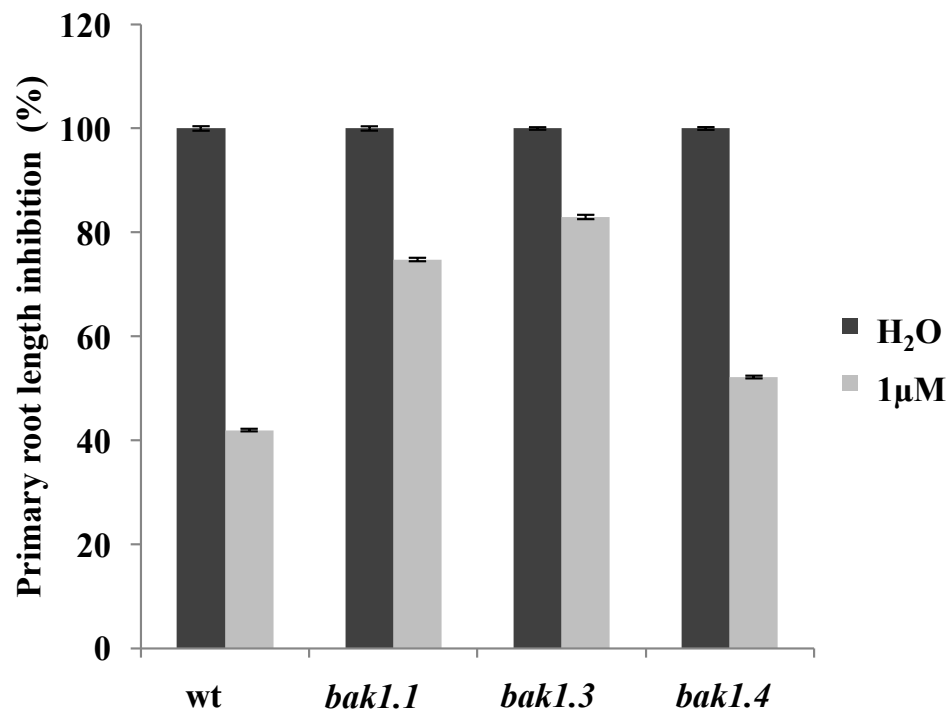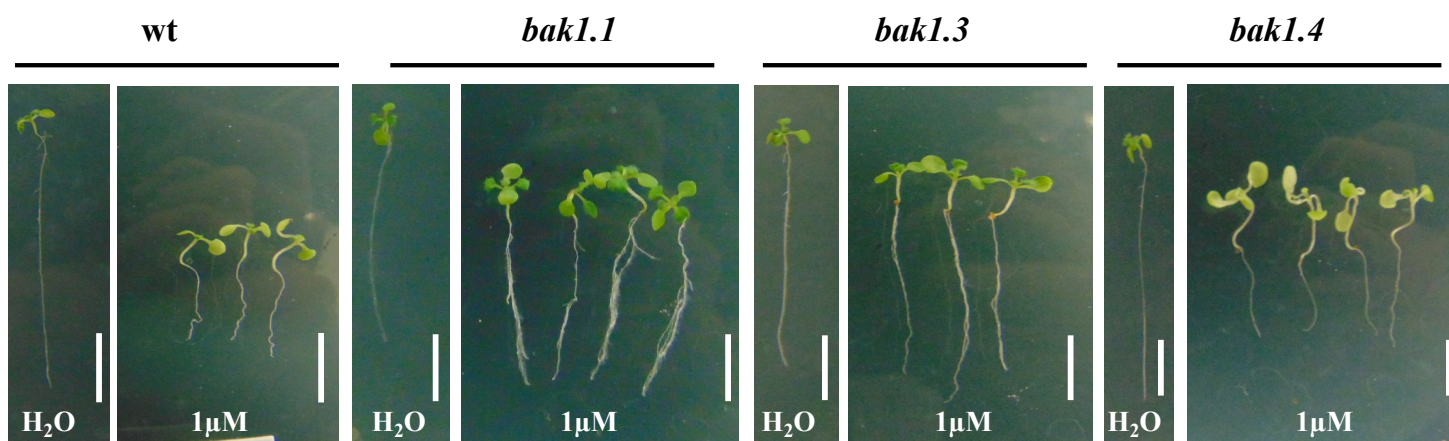

Supplement: S8 Fig — Four-day-old seedlings were transferred to medium containing 0 or 1μM BL, and the primary root length was measured after 8 days of treatment. The data represent the mean value of two experiments with +- SD of 15 and 10 seedlings in each one. Representative Arabidopsis seedlings after 8 days of treatment with BL are shown. The seedlings were arranged on plates after the treatment for imaging. Scale bars, 1cm. (PDF) [file pgen.1007053.s008.pdf]

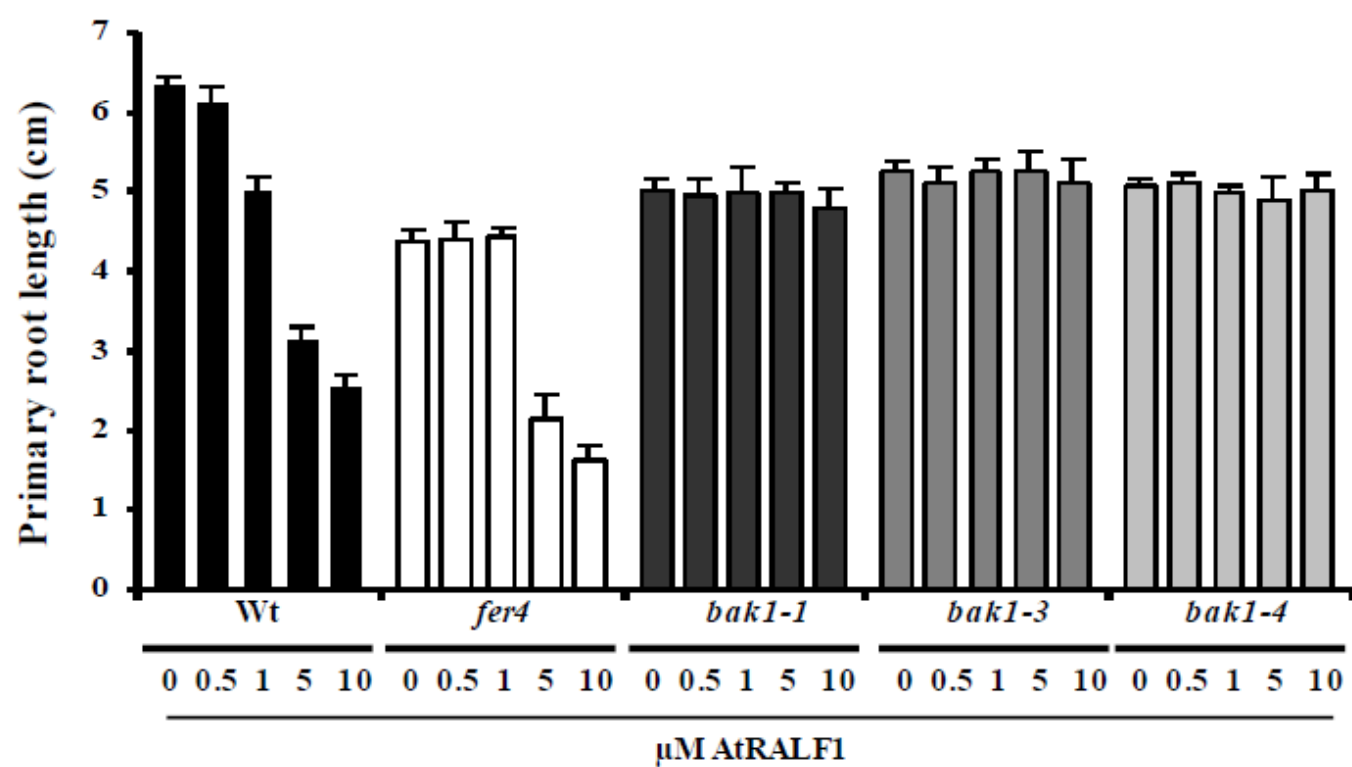

Supplement: S9 Fig — As opposed to fer4 mutant, that is insensitive to AtRALF1 peptide in low concentrations only, bak1 mutants are insensitive to AtRALF1 concentrations as high as 10 μM. Four-day-old seedlings were transferred to media containing different concentrations of AtRALF1, and the primary root length was measured after 8 days of treatment. The data represent the mean value ± SD of 30 seedlings. (PDF) [file pgen.1007053.s009.pdf]

A

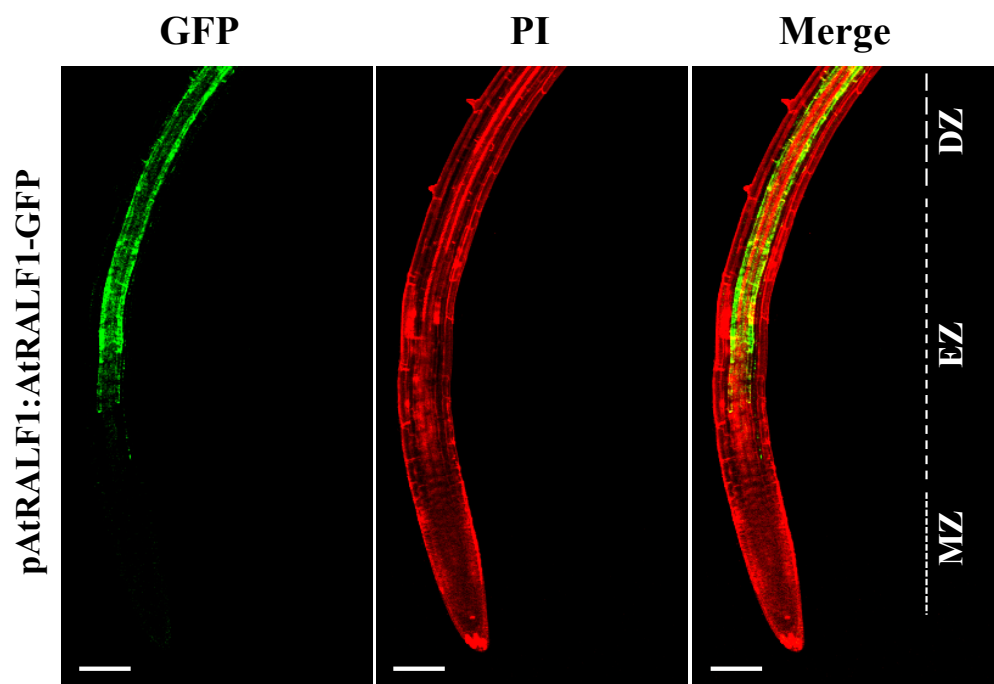

B

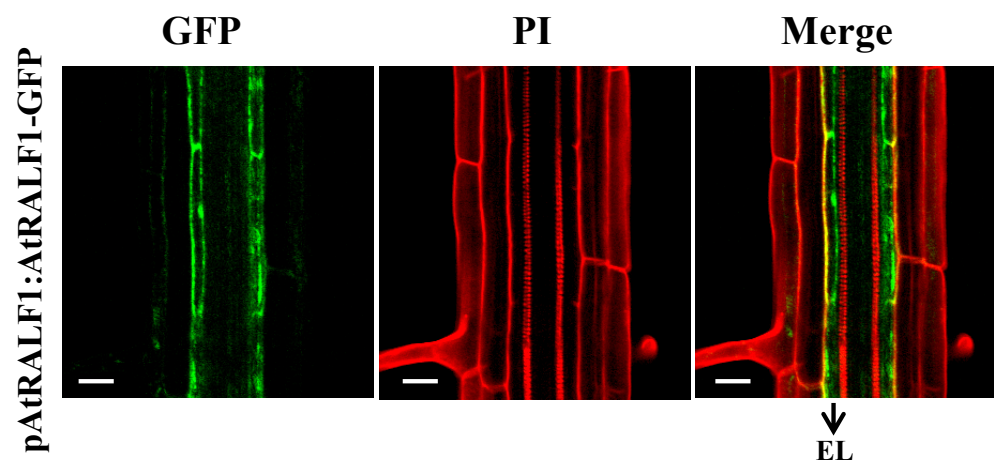

Supplement: S10 Fig — Confocal microscopic analysis of AtRALF1 peptide fused to GFP in roots of pAtRALF1:AtRALF1-GFP transgenic plants. (A) AtRALF1-GFP expression in the meristematic zone (MZ), elongation zone (EZ) and differentiation zone (DZ). Bars, 100 μm. (B) AtRALF1-GFP expression in the endodermis layer (EL). Bars, 25 μm. PI, propidium iodide. (PDF) [file pgen.1007053.s010.pdf]

A

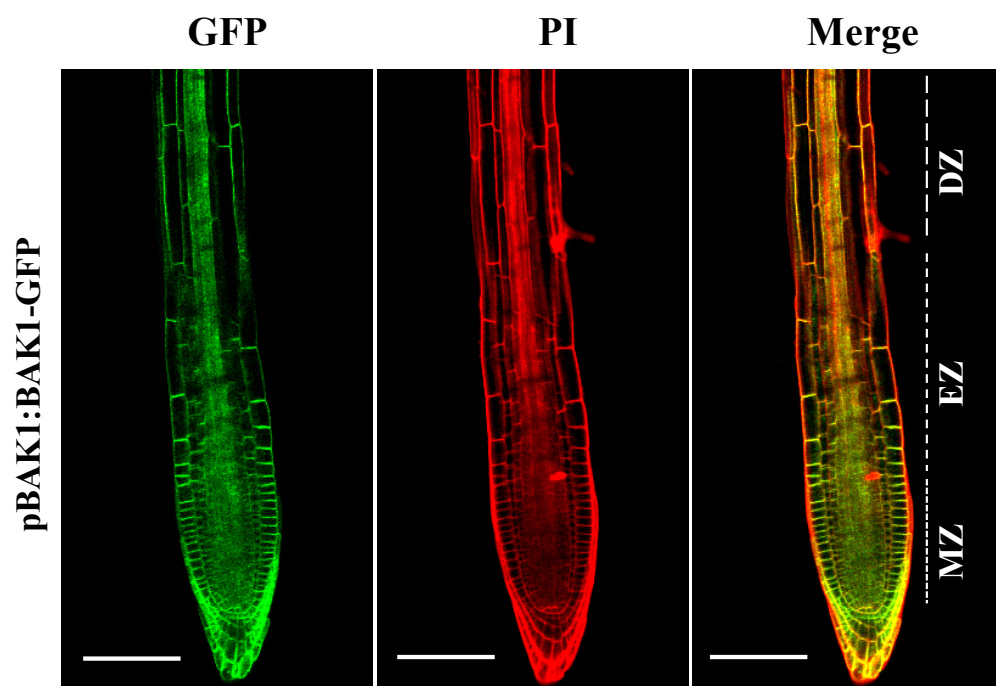

B

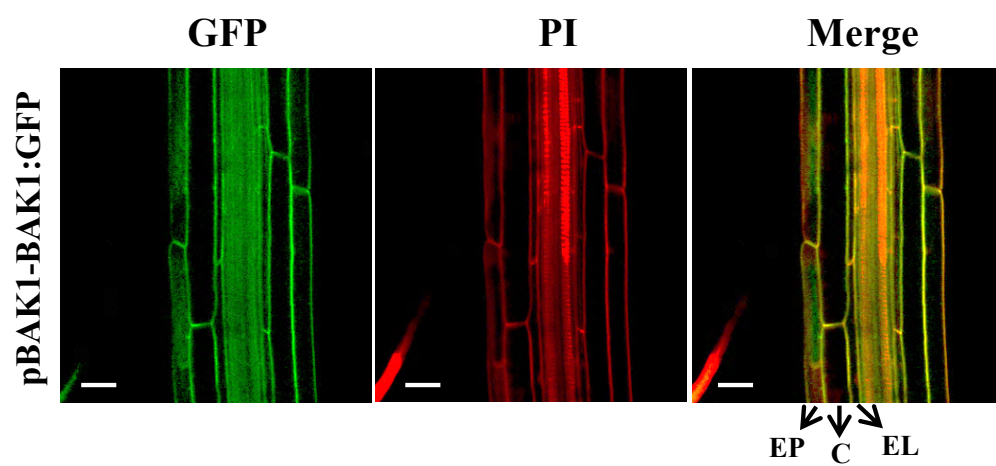

Supplement: S11 Fig — Confocal microscopic analysis of BAK1 peptide fused to GFP in roots of pBAK1:AtBAK1-GFP transgenic plants. (A) BAK1-GFP expression in the meristematic zone (MZ), elongation zone (EZ) and differentiation zone (DZ). Bars, 100 μm. (B) BAK1-GFP expression in endodermis layer (EL), cortex (C) and epidermis layer (EP). Bars, 25 μm. PI, propidium iodide. (PDF) [file pgen.1007053.s011.pdf]

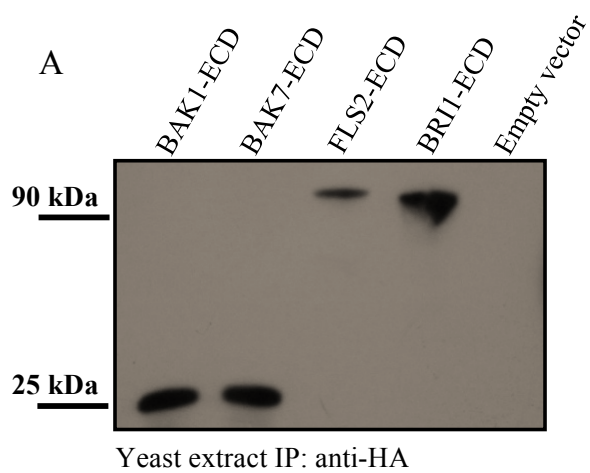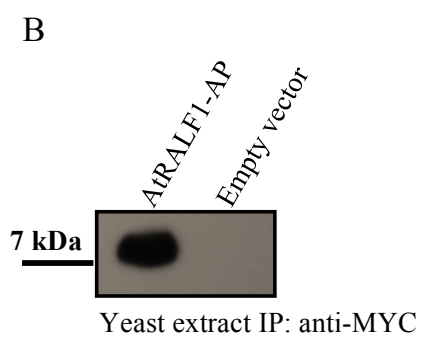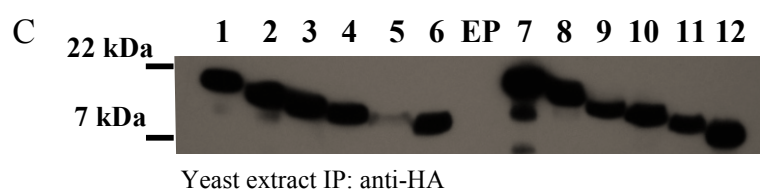

Supplement: S12 Fig — (A) Blot of HA-tagged proteins: BAK1-ECD:HA, BAK7-ECD:HA, BRI1-ECD:HA, and FLS2-ECD:HA. ECD, extracellular domain. The proteins were monitored using an anti-HA antibody. (B) Blot of the MYC-tagged protein AtRALF1 using an anti-MYC antibody. (C) Blot of HA-tagged truncated proteins: 1, LRR1 + LRR2 + LRR3 + LRR4 + LRR5 + Prorich; 2, LRR2 + LRR3 + LRR4 + LRR5 + Prorich; 3, LRR3 + LRR4 + LRR5 + Prorich; 4, LRR4 + LRR5 + Prorich; 5, LRR5 + Prorich; 6, Pro-rich; 7, Leu zippers + LRR1 + LRR2 + LRR3 + LRR4 + LRR5; 8, Leu zippers + LRR1 + LRR2 + LRR3 + LRR4; 9, Leu zippers + LRR1 + LRR2 + LRR3; 10, Leu zippers + LRR1 + LRR2; 11, Leu zippers + LRR1; 12, Leu zippers. The truncated proteins were monitored using an anti-HA antibody. EP, empty vector. Empty vectors were used as negative controls. (PDF) [file pgen.1007053.s012.pdf]

A

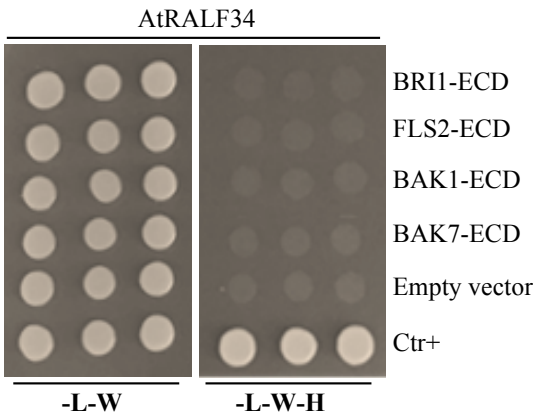

B

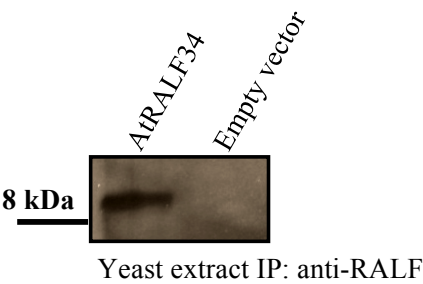

Supplement: S13 Fig — (A) The active AtRALF34 peptide fused to the GAL4 DNA-binding domain (AtRALF34-pGBKT7) was used as bait to test its interaction with the extracellular domain (ECD) of the proteins fused to the GAL4 activation domain (pGADT7): BAK1 (BAK1-ECD), BAK7 (BAK7-ECD), BRI1 (BRI1-ECD) and FLS2 (FLS2-ECD). Transformed yeast cells were selected on a synthetic complete medium lacking leucine, tryptophan and histidine to test interactions. (B) Analyses of protein extract from yeast: blot of the protein AtRALF34 using an anti-RALF antibody. The empty vector was used as negative control. (PDF) [file pgen.1007053.s013.pdf]

A

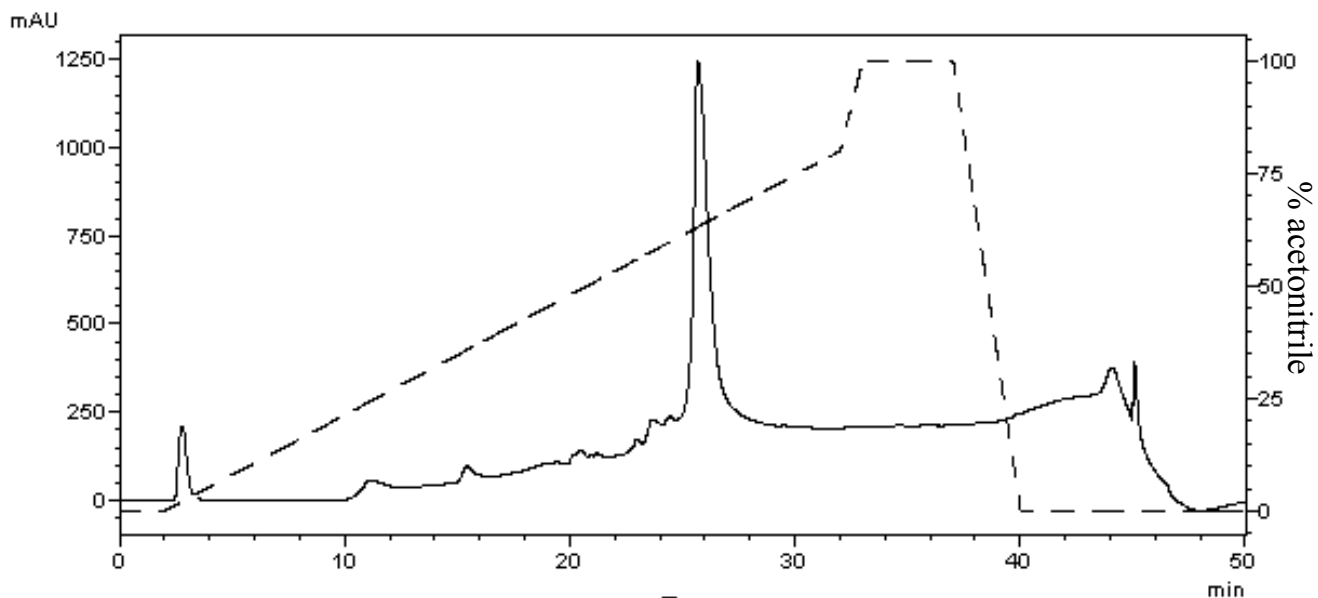

B

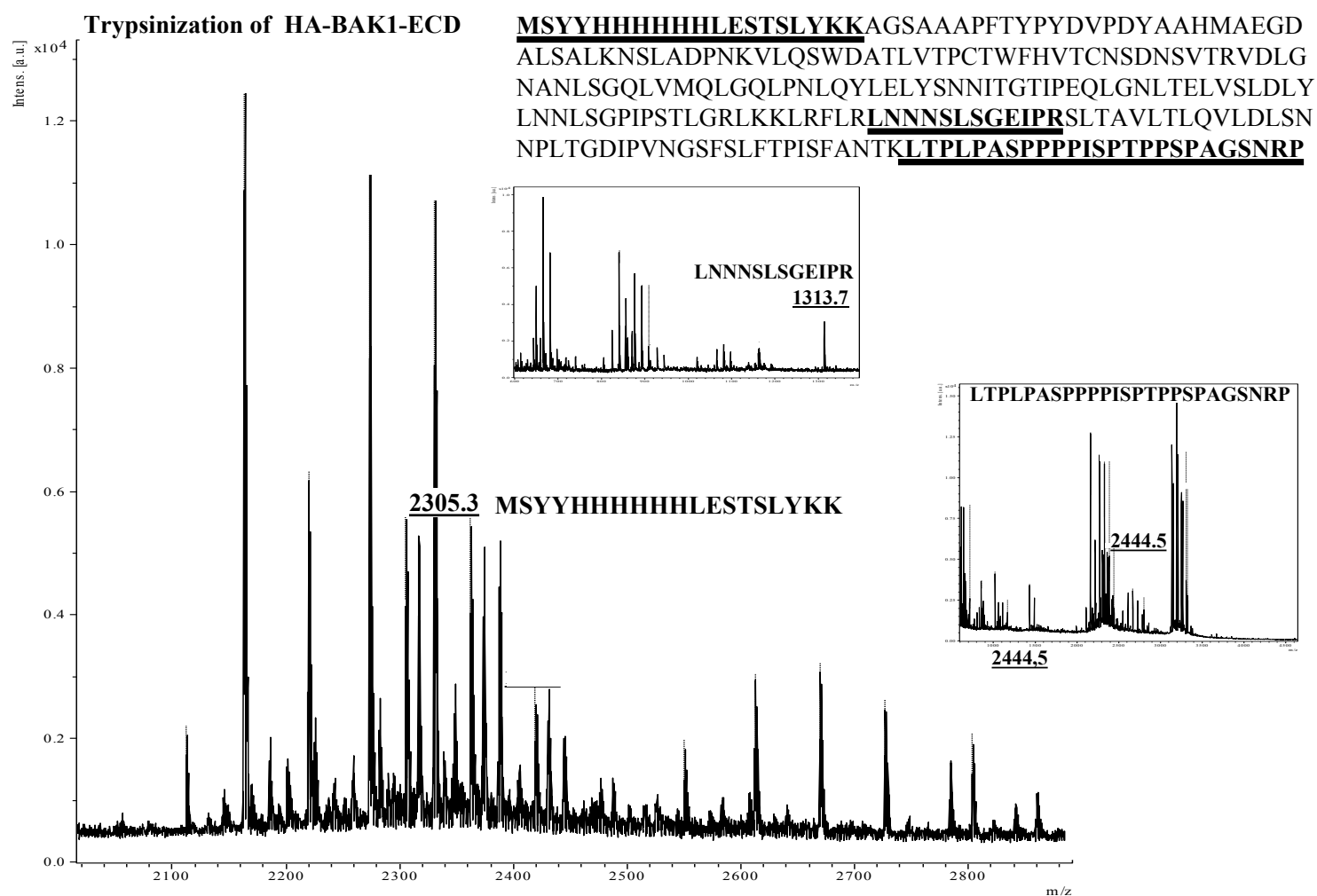

Supplement: S14 Fig — (A) The chromatographic profile of HA-BAK1-ECD recombinant protein eluted from a C-18 narrow-bore HPLC column. Dashed line shows the percentage of acetonitrile during the analysis. Approximately 50 μg of the protein was used. (B) Mass spectrometric analysis of trypsin-digested HA-BAK1-ECD protein. The mass of three tryptic fragments (1313.7; 2305.3 e 2444.5) obtained confirmed the identity of the peptide. The theoretical sequences are presented and underlined sequences indicate the identified fragments. No alterations in the HA-BAK1-ECD protein were detected. (PDF) [file pgen.1007053.s014.pdf]

A

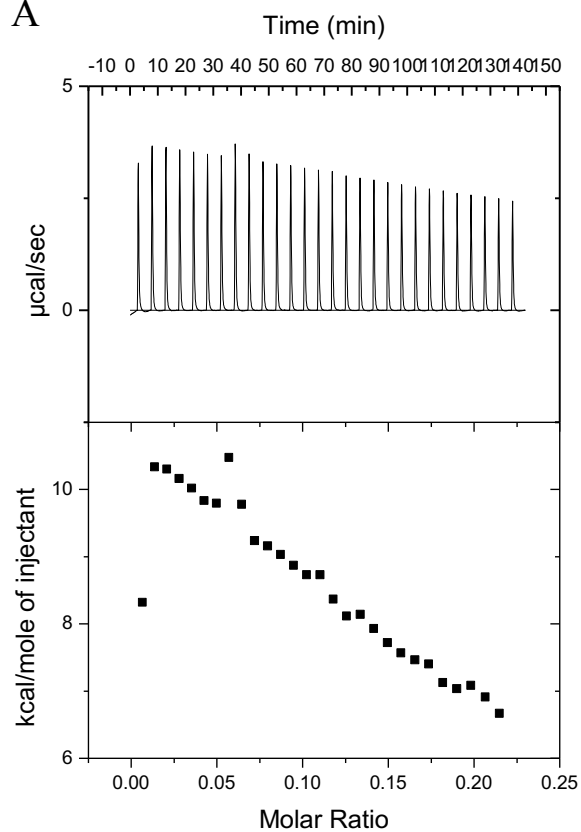

B

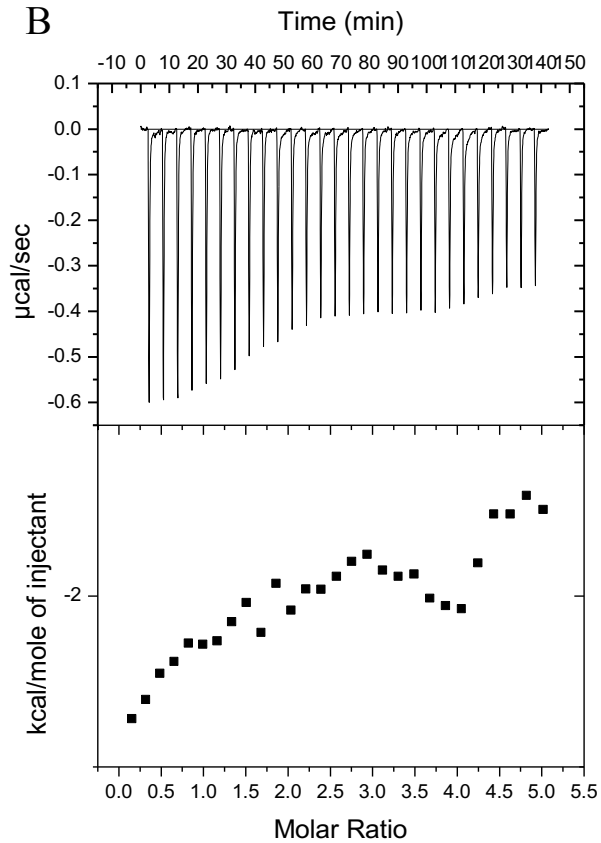

C

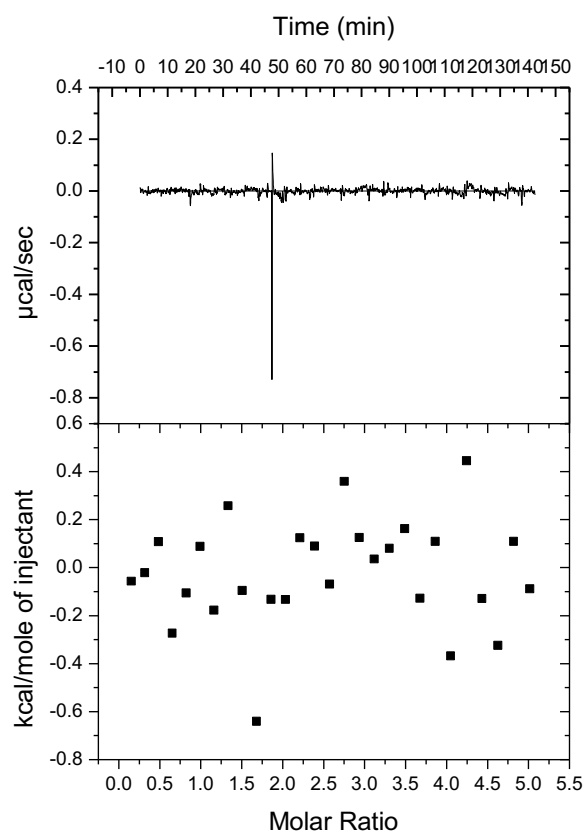

Supplement: S15 Fig — (A) AtRALF1 in 15mM HEPES, pH 6.8. ITC experiments were simulated using the following parameters: 28 injections of AtRALF1 (0.7 mM, volume 10 μL) in HEPES (volume cell = 1.4 mL) at 25°C. (B) BAK1-ECD in 15mM HEPES, pH 6.8. ITC experiments were simulated using the following parameters: 28 injections of BAK1-ECD (0.7 mM, volume 10 μL) in HEPES (volume cell = 1.4 mL) at 25°C. (C) HEPES in AtRALF1. ITC experiments were simulated using the following parameters: 28 injections of buffer 15mM HEPES, pH 6.8 (volume 10 μL) in AtRALF1 (0.03 mM, volume cell = 1.4 mL) at 25°C. (PDF) [file pgen.1007053.s015.pdf]

A

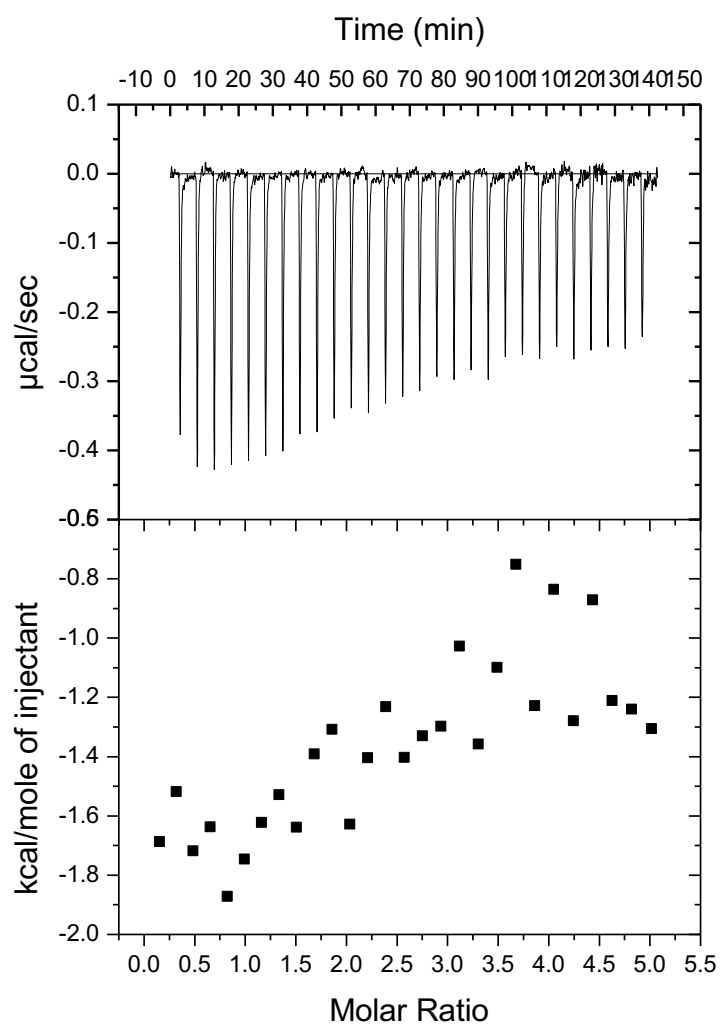

B

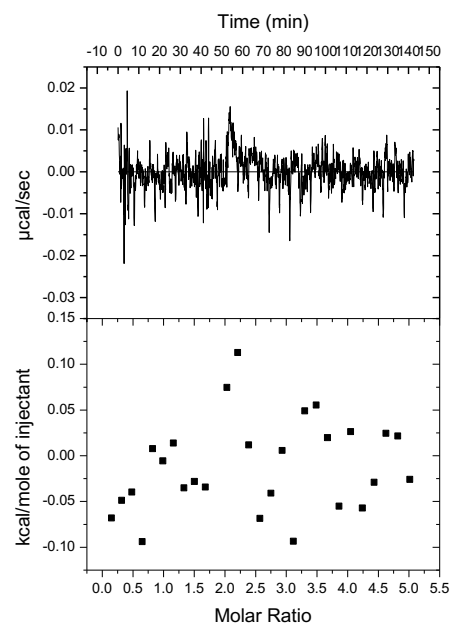

C

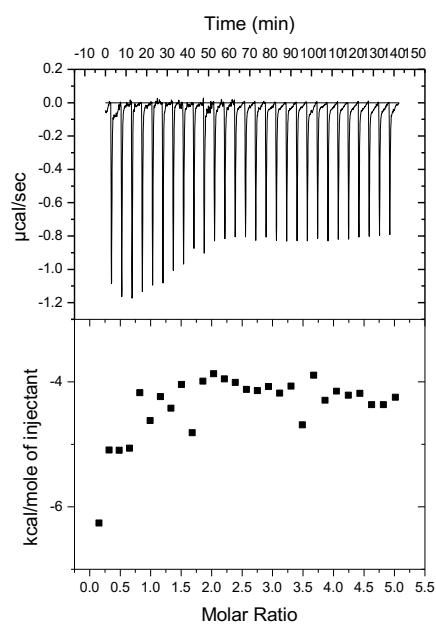

Supplement: S16 Fig — (A) BAK1-ECD in 0.03 mM RALF1(9–49). ITC experiments were simulated using the following parameters: 28 injections of BAK1-ECD (0.7 mM, volume 10 μL) in RALF1(9–49) (0.03 mM, volume cell = 1.4 mL) at 25°C. (B) HEPES in RALF1(9–49). ITC experiments were simulated using the following parameters: 28 injections of buffer 15mM HEPES, pH 6.8 (volume 10 μL) in RALF1(9–49) (volume cell = 1.4 mL) at 25°C. (C) BAK1-ECD in 15mM HEPES, pH 6.8. ITC experiments were simulated using the following parameters: 28 injections of BAK1-ECD (0.7 mM, volume 10 μL) in HEPES (volume cell = 1.4 mL) at 25°C. (PDF) [file pgen.1007053.s016.pdf]

A

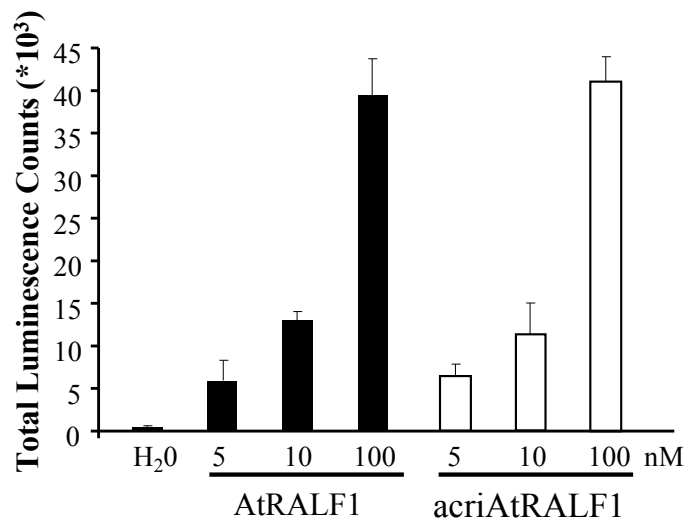

B

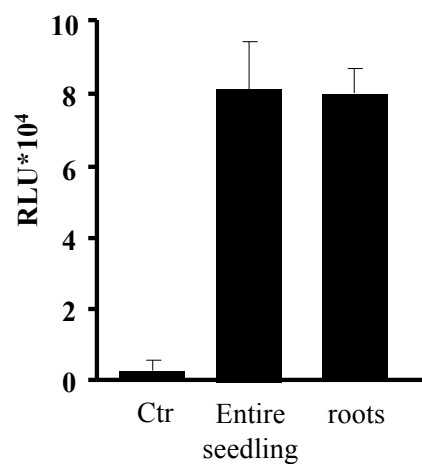

C

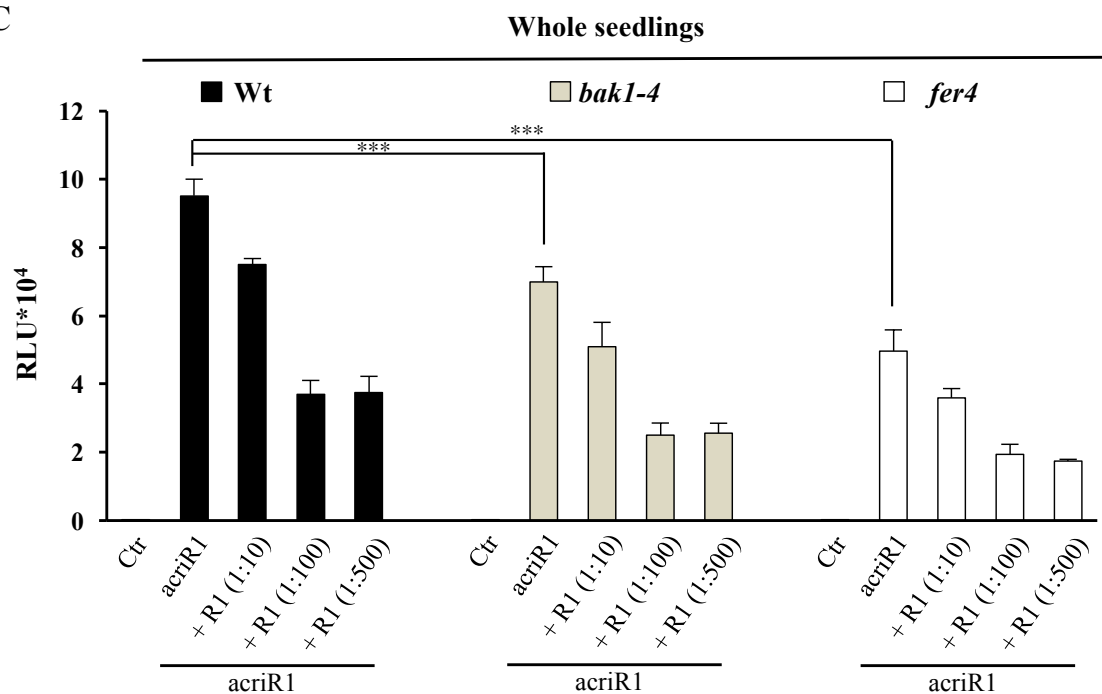

Supplement: S17 Fig — (A) The Ca2+ mobilization assay comparing the acriAtRALF1 activity with the AtRALF1 peptide. A total of 20 time-points per treatment were measured over 160 sec and summed. The results are the means ± SD of two measurements. (B) acriAtRALF1 binding in whole seedlings and only in roots of Arabidopsis. The roots were excised just before measurements. Ctr, seedlings treated with unlabeled AtRALF1. (C) Whole seedlings (5 days old) were treated for 20 min with acriAtRALF1 (acriR1), acriR1 and an excess of unlabeled AtRALF1 (R1), as indicated. The values are the mean ± SD of three measurements (5 seedlings each). The proportions of labeled: unlabeled proteins are shown in parentheses. Triple asterisks indicates P < 0.001 (Student’s t test). (PDF) [file pgen.1007053.s017.pdf]

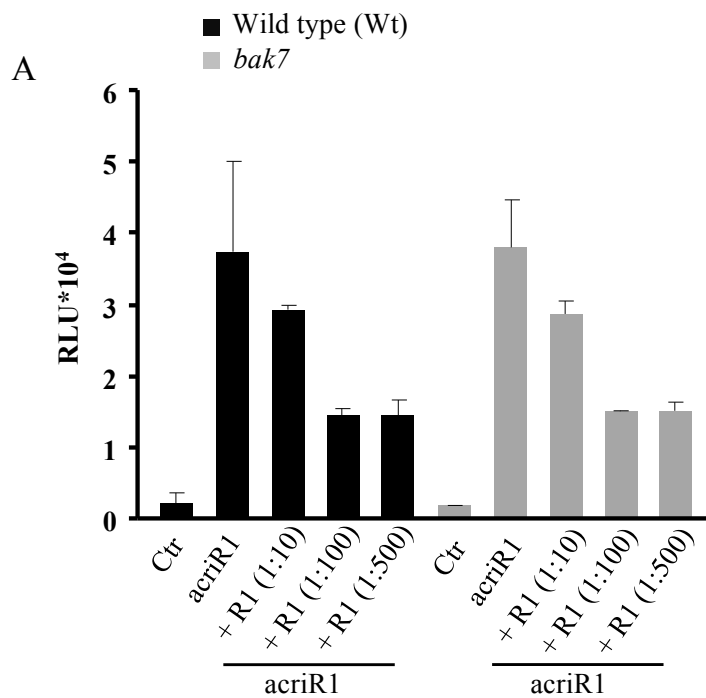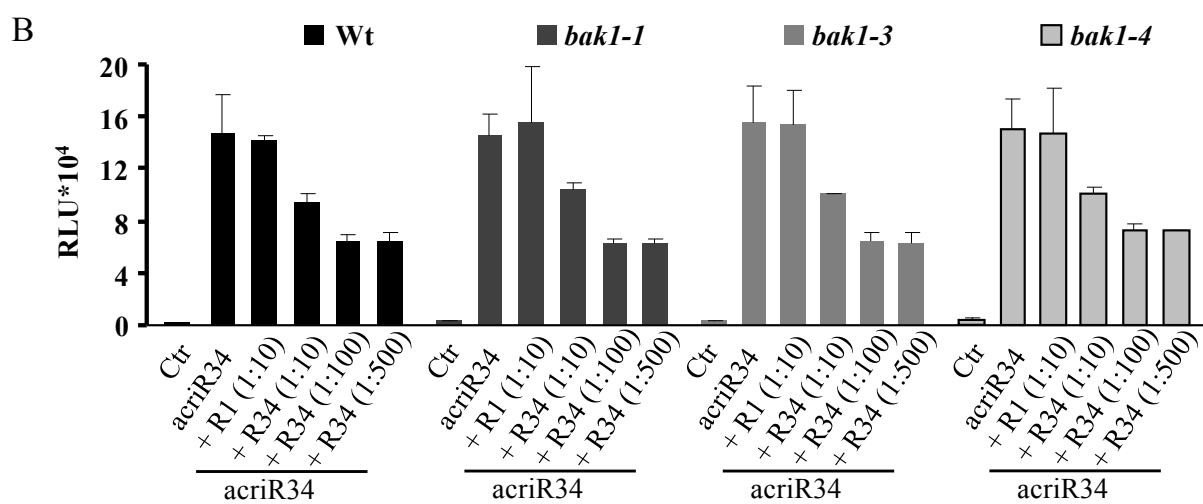

Supplement: S18 Fig — (A) bak7 seedlings (5 days old) were treated for 15 min with acriAtRALF1 (acriR1), acriR1 and an excess of unlabeled AtRALF1 (R1), as indicated. (B) bak1 seedlings (5 days old) were treated for 15 min with acriAtRALF34 (acriR34), acriR34 and an excess of unlabeled AtRALF34 (R34) or AtRALF1 (R1), as indicated. The proportions of labeled: unlabeled proteins are shown in parentheses. The values are the mean ± SD of two measurements (5 seedlings each). (PDF) [file pgen.1007053.s018.pdf]

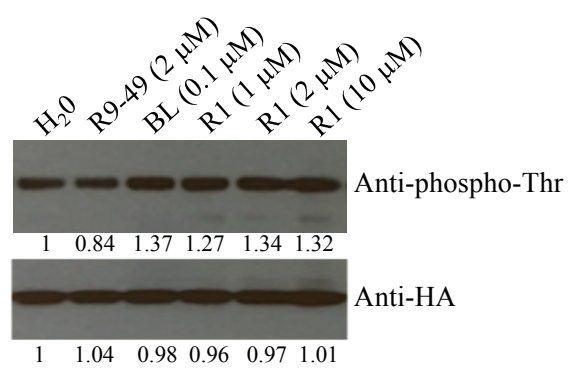

Supplement: S19 Fig — pBAK1:BAK1-6xHA plants were treated with water (H20), 2 μM RALF1(9–49) [R9-49], 0.1 μM BL, 1, 2 and 10 μM AtRALF1 for 20 min. After treatment, the microsomal fraction was isolated from roots. BAK1 protein was immunoprecipitated with anti-HA beads and subjected to immunoblot analysis with anti-phospho-Thr and anti-HA antibodies. The numbers indicate the relative intensity of the bands. (PDF) [file pgen.1007053.s019.pdf]

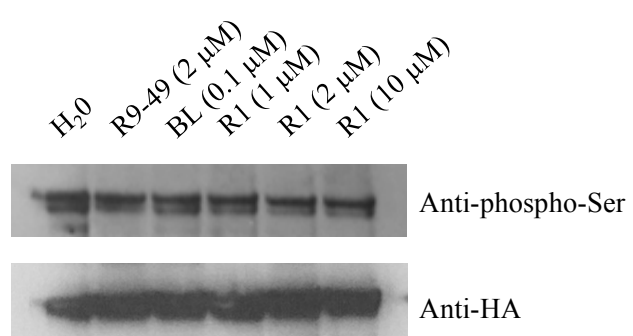

Supplement: S20 Fig — pBAK1:BAK1-6xHA plants were treated with water (H20), 2 μM RALF1(9–49) [R9-49], 0.1 μM BL, 1, 2 and 10 μM AtRALF1 for 20 min. After treatment, the microsomal fraction was isolated from roots. BAK1 protein was immunoprecipitated with anti-HA beads and subjected to immunoblot analysis with anti-phospho-Ser and anti-HA antibodies. (PDF) [file pgen.1007053.s020.pdf]

A

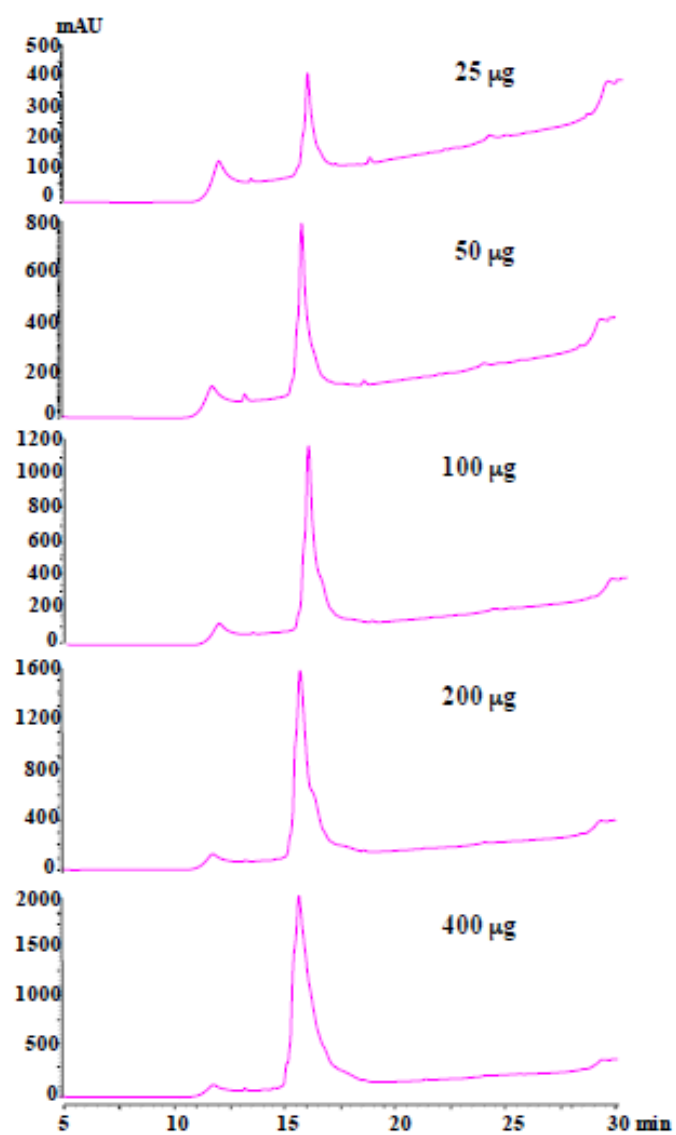

B

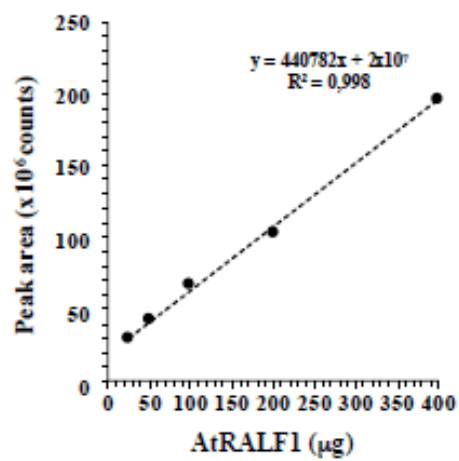

Supplement: S21 Fig — (A) HPLC profiles of the recombinant AtRALF1. The recombinant peptide was extracted from E. coli, affinity purified with a Ni2+ resin, dialyzed against 0.1% formic acid, HPLC purified, lyophilized, resuspended (0.1% formic acid) and injected again into a C18 reversed-phase HPLC column (8-mm 4,6 x 25 cm Kromasil) previously equilibrated with formic acid 0.1%. Increasing concentrations (25, 50, 100, 200 e 400 mg) were loaded into the column and eluted using an acetonitrile gradient (0 to 50% in 30 min). (B) Linear regression between the peak area and the corresponding AtRALF1 concentration. The linear equation and the R2 coefficient is shown. (PDF) [file pgen.1007053.s021.pdf]
