# Supplementary material for: BAK1 is involved in AtRALF1-induced inhibition of root cell expansion
Source: PLoS Genet. 2017 Oct 13;13(10):e1007053. doi: 10.1371/journal.pgen.1007053 (PMC5656322; doi:10.1371/journal.pgen.1007053)
Supplement: S1 Table — (PDF) [file pgen.1007053.s022.pdf]

S1 Table. Primers used.

Primer Name	Sequence 5´ - 3´	Purpose
AtRALF1-Fw	GGGCATATGATATTGGCGACCACAAAATACAT	Cloning for yeast two hybrid
AtRALF1-Rv	TTGGGATCCGTCTAACTCCTGCAACGAGCAAT	
AtRALF34- Fw	CGCATATGTATTACATATCGTACGG	
AtRALF34- Rv	TTAGGATCCCTTCATCTATCTCCGGCA	
BAK7-ECD-Fw	GCGCATATGGCTGAAGGTGATGCTTTGACT	
BAK7-ECD-Rv	CGCGGATCCCCATTTGCCCCCCTGAAGGT	
BRI1-ECD-Fw	GCGCATATGGCTTCACCATCTCAGTCTTTA	
BRI1-ECD-Rv	CGCGGATCCCTCCCATCGCCACACTACCAGCAA	
FLS2-ECD-Fw	GCGCATATGAAACAGAGCTTTGAACCAGAGAT	
FLS2-ECD-Rv	CGCGGATCCCAATCAGGATGACTCTGGTT	
BAK1-ECD-Fw	GCGCATATGGCCGAAGGTGATGCTCTAA	
BAK1-ECD-Rv	CGCGGATCCCTCTATTACTCCCTGCAGGTGAT	
N-term-L zippers-Fw	GCGCATATGGACAATAGTGTTACACGTGT	
N-term-L zippers-Rv	CGCGGATCCCTCTATTACTCCCTGCAGGTGAT	
N-term-LRR1-Fw	GCGCATATGCTTCCAAACTTGCAGTACTTGGA	
Primer Name	Sequence 5´ - 3´	Purpose
N-term-LRR1-Rv	CGCGGATCCCTCTATTACTCCCTGCAGGTGAT	Cloning for yeast two hybrid
N-term-LRR2-Fw	GCGCATATGCTGACGGAATTGGTGAGCTT	
N-term-LRR2-Rv	CGCGGATCCCTCTATTACTCCCTGCAGGTGAT	
N-term-LRR3-Fw	GCGCATATGCTTAAGAAACTCCGTTTCTTGCGT	
N-term-LRR3-Rv	CGCGGATCCCTCTATTACTCCCTGCAGGTGAT	
N-term-LRR4-Fw	GCGCATATGGTCCTGACGCTACAAGTTCT	
N-term-LRR4-Rv	CGCGGATCCCTCTATTACTCCCTGCAGGTGAT	
N-term-LRR5-Fw	GCGCATATGTCACTTTTCACTCCAATCAGTT	
N-term-LRR5-Rv	CGCGGATCCCTCTATTACTCCCTGCAGGTGAT	
C-term-P rich region-Fw	GCGCATATGGCCGAAGGTGATGCTCTAAGT	
C-term-P rich region-Rv	CGCGGATCCCAAAGGAACCATTAACAGGA	
C-term-LRR1-Fw	GCGCATATGGCCGAAGGTGATGCTCTAAGT	
C-term-LRR1-Rv	CGCGGATCCCGCTATTGCAAGTAACATGAA	
C-term-LRR2-Fw	GCGCATATGGCCGAAGGTGATGCTCTAAGT	
C-term-LRR2-Rv	CGCGGATCCCCTGACCAAGTTGCATTACGAGCT	
Primer Name	Sequence 5´ - 3´	Purpose
C-term-LRR3-Fw	GCGCATATGGCCGAAGGTGATGCTCTAAGT	Cloning for yeast two hybrid
C-term-LRR3-Rv	CGCGGATCCCATTTCCAAGCTGTTCTGGGA	
C-term-LRR4-Fw	GCGCATATGGCCGAAGGTGATGCTCTAAGT	
C-term-LRR4-Rv	CGCGGATCCCTCGGCCGAGAGTTGATGGAAT	
C-term-LRR5-Fw	GCGCATATGGCCGAAGGTGATGCTCTAAGT	
C-term-LRR5-Rv	CGCGGATCCCAGCAGTCAAAGACCTTGGA	
GAPDH-Fw	TTGGTGACAACAGGTCAAGCA	Semi-quantitative RT-PCR and qRT-PCR
GAPDH-Rv	AAACTTGTCGCTCAATGCAATC	
PRP3-Fw	GTTCCGACCCAGCATCATAC	
PRP3-Rv	GCAAGTCTCGACCGGAGATA	
HRGP2-Fw	CACAGTTGCTAATGGAAACGA	
HRGP2-Rv	CGGCGGTGAATATCTCTTGT	
CPD-Fw	AGCAACTCGGTAACGACAGG	
CPD-Rv	CAGAGAGTGCAACCCTAGCC	
DWF4-Fw	GTGGGTGGAAAGTGTTACCG	
DWF4-Rv	CTGTTGCCATCTCCAAGGAT	
BAK1-Fw	GTCAGAAAGTAGTGTCGCCA	
BAK1-Rv	ACTTGTAGCGTCAGGACAGC	
AtRALF1- Fw	GGGCATATGATATTGGCGAC	
AtRALF1- Rv	TTGGGATCCGTCTAACTCCT	
Promoter 35S - Fw	ATCCCACTATCCTTCGCAAGACCCTTCCTCTA	
Aequorin-Rv	TGGTGTATGCTTTCCATTCATCCA	
